# The Apical Complex Provides a Regulated Gateway for Secretion of Invasion Factors in *Toxoplasma*

**DOI:** 10.1371/journal.ppat.1004074

**Published:** 2014-04-17

**Authors:** Nicholas J. Katris, Giel G. van Dooren, Paul J. McMillan, Eric Hanssen, Leann Tilley, Ross F. Waller

**Affiliations:** 1 School of Botany, The University of Melbourne, Parkville, Victoria, Australia; 2 Research School of Biology, Australian National University, Canberra, Australian Capital Territory, Australia; 3 Biological Optical Microscopy Platform, The University of Melbourne, Parkville, Victoria, Australia; 4 Advanced Microscopy Facility, The University of Melbourne, Parkville, Victoria, Australia; 5 Department of Biochemistry and Molecular Biology, Bio21 Molecular Science and Biotechnology Institute, and Australian Research Council Centre of Excellence for Coherent X-ray Science, The University of Melbourne, Parkville, Victoria, Australia; 6 Department of Biochemistry, University of Cambridge, Cambridge, United Kingdom; Washington University School of Medicine, United States of America

## Abstract

The apical complex is the definitive cell structure of phylum Apicomplexa, and is the focus of the events of host cell penetration and the establishment of intracellular parasitism. Despite the importance of this structure, its molecular composition is relatively poorly known and few studies have experimentally tested its functions. We have characterized a novel *Toxoplasma gondii* protein, RNG2, that is located at the apical polar ring—the common structural element of apical complexes. During cell division, RNG2 is first recruited to centrosomes immediately after their duplication, confirming that assembly of the new apical complex commences as one of the earliest events of cell replication. RNG2 subsequently forms a ring, with the carboxy- and amino-termini anchored to the apical polar ring and mobile conoid, respectively, linking these two structures. Super-resolution microscopy resolves these two termini, and reveals that RNG2 orientation flips during invasion when the conoid is extruded. Inducible knockdown of RNG2 strongly inhibits host cell invasion. Consistent with this, secretion of micronemes is prevented in the absence of RNG2. This block, however, can be fully or partially overcome by exogenous stimulation of calcium or cGMP signaling pathways, respectively, implicating the apical complex directly in these signaling events. RNG2 demonstrates for the first time a role for the apical complex in controlling secretion of invasion factors in this important group of parasites.

## Author Summary

Apicomplexan parasites comprise major human pathogens, including the malaria-causing parasites *Plasmodium* spp., and *Toxoplasma gondii* that causes birth defects and neurological disorders. Key to the success of this group was the evolution of the apical complex, a structure at the focus of the events of host cell invasion. This structure was recently shown to derive from elements of the flagellar apparatus, and rudiments of an apical complex are used for feeding in related protists. Evolution of host cell invasion in Apicomplexa has entailed development of a coordinated secretion of invasion factors from the cell apex. Little is known, however, of the behaviour or function of the components of the apical complex during invasion. We have characterized a new protein, RNG2, that forms a ring at the heart of the apical complex in *T. gondii*. This is a dynamic ring that links the mobile conoid with the apical polar ring, and is assembled as one of the first structures in replicating parasites. When RNG2 is artificially depleted, cells become insensitive to the molecular cues for secretion, and invasion of host cells is blocked. This reveals that the apical complex participates directly in regulating secretion, and controlling the events of invasion.

## Introduction

Apicomplexans are obligate parasites of metazoans that non-destructively enter their host cells. Here they feed and replicate before destructively escaping in search of further cells to invade. Apicomplexa comprises over 6000 species that parasitize virtually every animal group [1]. The malaria-causing parasites, *Plasmodium* spp., are best known for their pattern of invasion and release from human red blood cells, causing cyclic fevers and the symptoms of malaria that annually result in 0.6 to 1 million deaths per year and morbidity in up to 220 million people [2]. *Toxoplasma gondii* can infect most nucleated mammalian cell types and infects approximately one third of the human population. Human infections are typically relatively asymptomatic, however *T. gondii* causes acute and even fatal disease in immuno-compromised individuals (encephalitis and ocular disease), severe or lethal developmental defects in unborn fetuses, and significant agricultural losses through miscarriage in livestock [3]. Early-diverging apicomplexans (gregarines) are limited to invertebrate hosts and their invasion is incomplete, with feeding often achieved through the apical tip of the parasite being intimately buried within the host cell [4], [5].

**Figure 1:**
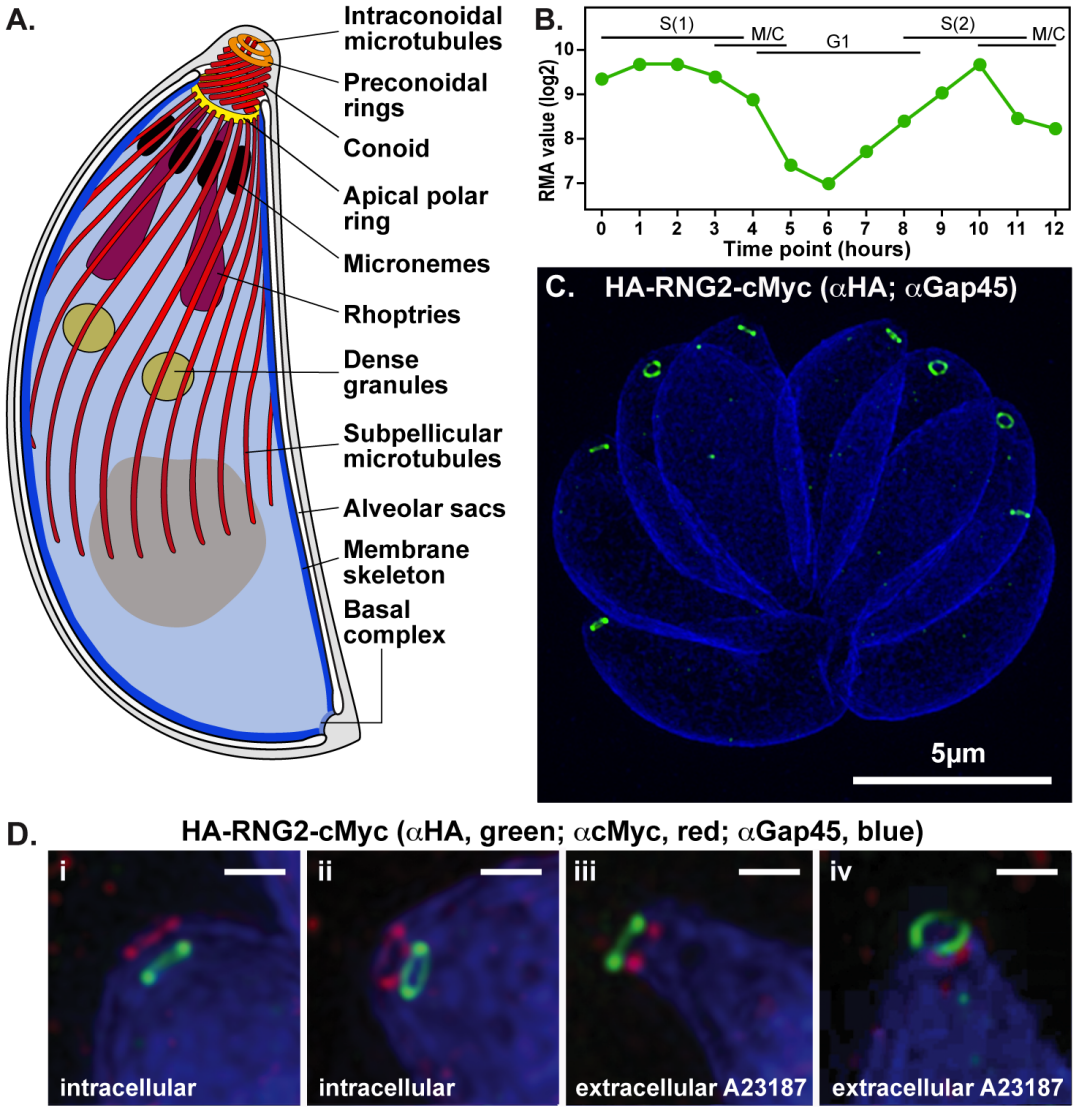
RNG2 apical rings. (A) Schematic of *Toxoplasma gondii* cell representing the structural elements of the apical complex, the cell pellicle, and the secretory organelles. The conoid is shown extruded. (B) RNG2 expression throughout the cell cycle based on dataset of Behnke et al. [31]. (C) 3D-SIM image of intracellular HA-RNG2-cMyc parasites showing the RNG2 N-terminus (HA, green) against GAP45 labeling of the IMC (blue). (D). 3D-SIM images of intracellular parasites with retracted conoids (i, ii) and extracellular parasites treated with A23187 to extrude conoids (iii, iv) labeled for RNG2 N-terminus (green) and C-terminus (red), and GAP45 (blue). Scale bar = 500 nm.

The defining feature of Apicomplexa is a complex assemblage of structural and secretory elements at the apical point of the cell, forming the namesake of the group—the apical complex. The apical complex is instrumental in the host cell invasion processes [6], [7]. It provides both a semi-rigid framework to these apically pointed cells, and a focal point for secretory organelles that release various invasion factors that mediate interaction with, and invasion of, the host cell. The apical complex is organized around an apical polar ring that serves as a microtubule organizing center that nucleates an array of subpellicular microtubules that descend toward the posterior of the cell (Figure 1A) [8]–[10]. These microtubules subtend flattened membrane sacs, or alveoli, that line most of the plasma membrane [11]. A fibrous proteinaceous membrane skeleton supports the alveolar sacs against the microtubules [12]. The alveoli and proteinaceous skeleton form a structure called the inner membrane complex (IMC), which, together with the subpellicular microtubules, provides the shape and stability of the cell. The apical polar ring marks the apical extremity of the IMC. A mobile conoid, consisting of tightly bent tubulin filaments fused to form a tapered hollow barrel, sits within the apical polar ring [10], [11], [13]. The conoid can either be recessed in the cell, so that its tip is flush with the apical polar ring, or, during invasion, be extruded from the apical polar ring to form an extended point to the cell (Figure 1A). At the tip of the conoid are two preconoidal rings, and a pair of short microtubules sit eccentrically within the conoid. These preconoidal rings and interconoidal microtubules move together with the conoid during extrusion [8].

The structural elements of the apical complex provide orientation to the cell, and are the focal point for arrays of secretory organelles—micronemes and rhoptries—that cluster towards the base of the conoid in readiness for a staged sequence of release (Figure 1A) [14]. Microneme contents are secreted first, prior to invasion, and coat the parasite with proteins that facilitate host cell adhesion, gliding motility, and contribute to formation of an annular moving junction with the host plasma membrane through which the parasite enters the host. During invasion rhoptries secrete further elements of the moving junction, as well as proteins that establish the properties of the parasitophorous vacuole within which the parasite typically resides.

The elements of the apical complex are highly conserved throughout Apicomplexa, although secondary reduction is evident. For example, the conoid is only intermittently present within various members and life stages of haemosporins (including *Plasmodium* spp.) and likely completely lost from piroplasms such as *Babesia* and *Theileria* [15], [16]. The presence of the apical polar ring, however, is seemingly universal. Apicomplexans also have a distinctive model of cell division, whereby daughter cells form within a mother cell. The pellicle, consisting of the alveolar sacs and protein skeleton (and plasma membrane in mature cells), with the associated subpellicular microtubules, is amongst the first structures formed in the new daughter cells, and this lays down the scaffold for nuclei and organelles to correctly partition into these daughters [17]–[22]. Markers for the conoid in *T. gondii* also appear early in daughter cell formation [23], suggesting the apical complex is also formed early in this process. The apical complex, therefore, likely plays pivotal roles in both cell division and host cell invasion.

Despite extensive characterization of the apical complex through ultrastructural studies, there is relatively limited knowledge of the molecular composition of its structural elements, and even less experimental illumination of its function. *T. gondii* provides the best studied system to date, with several proteins that associate with the apical complex structures identified either through proteomics or reporter protein tagging. The conoid itself is composed of tubulin, and is known to be decorated with several proteins (*Tg*Centrin3; calcium-binding domain proteins CAM1 and CAM2; dynein light chain, *Tg*DLC), although the functions of these proteins remain untested [13], [23]. The preconoidal rings are associated with *Tg*Centrin2 and SAS6L, two proteins typically implicated with centriolar function [23]–[25]. SAS6L knockout cells showed a subtle negative growth phenotype, however the basis of this phenotype has not been determined [24]. The novel protein TgICMAP1 decorates the intraconoidal microtubules but its function is unknown [26]. Even the curious behavior of conoid extrusion, while hypothesized to provide some mechanical role in invasion, has evaded clear insight into its function [27]. A small number of proteins associated with the apical cap of the IMC are closely associated with the apical complex, but these appear to serve more general pellicle functions in gliding motility (GAP70), coordination of assembly and spatial organization of the pellicle (IMC15, ISP1, MORN1) or are of unknown function (PhIL1, *Tg*DLC) [18]. The composition of the apical polar ring is the most poorly characterized of these structures, despite its central role and universality in the phylum. A single protein, RNG1, has been localized to this structure [28]. RNG1 associates with the apical polar ring only as daughter cells reach maturity, so presumably is not responsible for the early formation of this ring. Attempts to generate a knockout were unsuccessful, suggesting an essential but undetermined function.

As part of a broader study of conserved pellicle proteins of Infrakingdom Alveolata (Apicomplexa, Dinoflagellata, Ciliophora), we previously identified a novel *T. gondii* protein that localized as a ring in the region of the apical complex [29]. To investigate the function of the apical complex we have investigated the localization and behavior of this ring protein (we now call RNG2) during cell replication and invasion, and examined its role by inducible knockdown of RNG2 expression. These results confirm that new components of the apical complex are first assembled at the centrosomes at the earliest stages of cell replication, and that the apical complex acts as a gatekeeper that regulates secretion during parasite invasion.

## Results

We previously identified *T. gondii* pellicle protein TGME49_244470 (toxodb.org) through its similarity to uncharacterized proteins found in the cell pellicles of the related phylum Ciliophora [29]. These proteins share repetitive protein sequences biased for lysine, glutamic acid, glutamine and hydrophobic residues (leucine, isoleucine and valine). TGME49_244470 localized to an apical ring structure, similar to a previously identified apical ring protein, RNG1 [28], [29]. We, therefore, name the new protein RNG2 although it shares no sequence similarity to RNG1. RNG2 expression is cell cycle-dependent, peaking during DNA synthesis and mitosis (Figure 1B) [30], [31], consistent with early appearance of RNG2 rings in developing daughter cells [29]. RNG2 is a large protein (2595 amino acid, 290 kDa), predicted to form multiple coiled-coils (from approximately amino acid 550 to 2150; COILS, [32]).

### RNG2 bridges the apical polar ring and conoid

To investigate the fine localization and interactions of RNG2 with other apical structures we used 3D-structured illumination microscopy (SIM), which provides an 8-fold increase in volume resolution over conventional light microscopy [33], [34]. Given the large size of RNG2 and its potential to fill a volume larger than SIM resolution, we separately tagged the N-terminus and C-terminus, with epitope tags HA and cMyc, respectively (HA-RNG2-cMyc, see knockdown construct below). Immuno-detection of these tags in intracellular parasites shows that each forms a continuous apical ring in mature cells measuring 380 +/− 20 nm (standard deviation (SD)) in diameter (Figure 1C, D). These two rings are consistently displaced, with the C-terminus (red) adjacent to the apical extremity of the GAP45-labeled IMC (blue), and the N-terminus (green) occurring below the IMC in the region of the retracted conoid (Figure 1Di and ii). This implies that RNG2 forms a tube or collar, with a consistent orientation of the protein termini. The conoid can also be extruded relative to the apical polar ring, a state that typically occurs during invasion. Conoid extrusion can be artificially achieved by exposure to the calcium ionophore A23187 [35], [36]. When extracellular parasites were treated with A23187 to effect conoid extrusion, the orientation of the RNG2 N- and C-terminus was completely reversed, with the N-terminus now extended anterior to the C-terminus (Figure 1Diii and iv). To test that the RNG2 protein termini could be reproducibly detected, we also created a RNG2 fusion with fluorescent proteins (mCherry-cMyc-RNG2-GFP). Observation of these live cell markers resulted in the same pattern of a polarized orientation of the RNG2 collar that inverted upon conoid extrusion (Figure S1).

**Figure 2:**
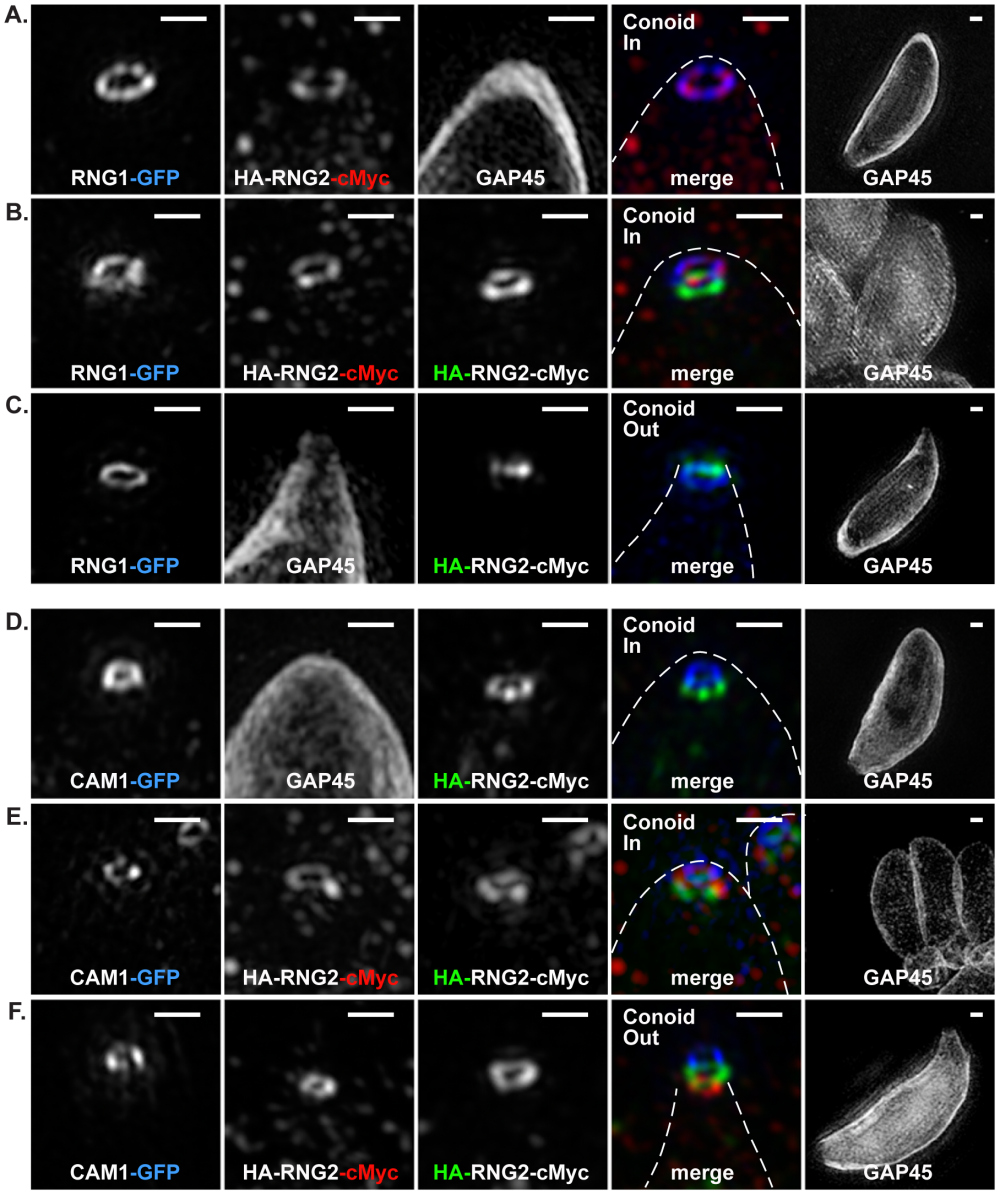
3D-SIM of RNG2 location relative apical polar ring marker RNG1 and conoid marker CAM1. (A-C) RNG1-GFP (pseudo-colored blue), and (D-F) CAM1-GFP (pseudo-colored blue) colocalized with HA (green) and cMyc (red) of the HA-RNG2-cMyc fusion. Conoid position is indicated. Individual immuno-signals are shown in monochrome, and colored in the merged image where GAP45 labeling is shown by a dashed outline. Right hand column shows GAP45 labeling of each cell at 0.25 magnification. Scale bars = 500 nm.

To better understand the RNG2 flip during conoid extrusion, the two termini were co-labeled with markers for the apical polar ring, RNG1 [28], and the conoid, CAM1 [23]. RNG1 was transiently expressed as a RNG1-GFP fusion in the HA-RNG2-cMyc cells. When the conoid was in the retracted position, the C-terminus of RNG2 (red) collocates with RNG1 (blue), whereas the RNG2 N-terminus (green) is posterior (Figure 2A and B). This indicates that the C-terminus of RNG2 is in close association with the apical polar ring. When the conoid is extruded by A23187, the N-terminus of RNG2 (green) is now extended anterior of RNG1 (blue), suggesting that it has passed through the apical polar ring (Figure 2C). CAM1 is a known conoid marker, although its precise location on the conoid remains undetermined [18], [23]. We transiently expressed CAM1-GFP in the HA-RNG2-cMyc cells to observe the position and behavior of the RNG2 termini with respect to this conoid marker. Using SIM the CAM1-GFP (blue) resolved as a narrow ring of smaller diameter than the apical polar ring, but of similar apparent depth (Figure 2D-F). The N-terminus of RNG2 was a consistent distance posterior to CAM1, maintaining this position when the conoid was extruded and RNG2 flipped. These data suggest that the C-terminus of RNG2 is attached to the apical polar ring, and the N-terminus is attached to the conoid at a position posterior to CAM1. During conoid extrusion and retraction the RNG2 N-terminus moves with the mobile conoid, while the C-terminus is apparently anchored to the apical polar ring. CAM1 itself is confined to a narrow band on the conoid, likely towards the conoid tip as when the conoid was retracted the CAM1 ring was approximately level with RNG2 C-terminus (Figure 2E).

### RNG2 is an early marker for daughter cell assembly

**Figure 3:**
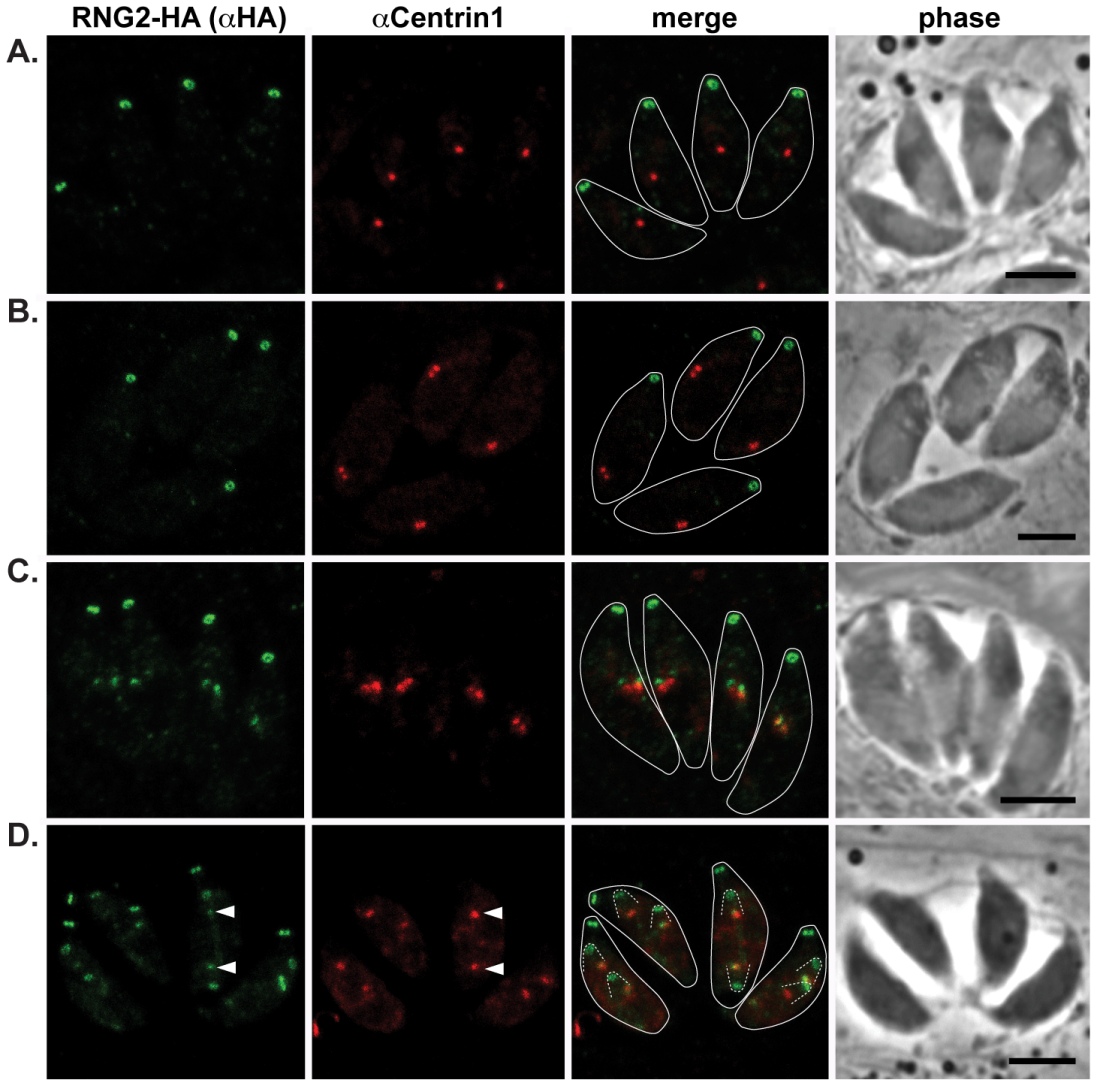
RNG2 appears in daughter cells after centrosome duplication. RNG2-HA (green) cells immuno-labeled for centrin1 (red) show single centrosome duplication at the beginning of daughter cell formation (A, B), after which RNG2 appears in association with each centrosome (C). (D) As centrosome pairs separate, RNG2 dissociates and forms rings, but leaves a trace of RNG2 in association with the centrosome (e.g. arrowheads). Inferred daughter buds shown with dashed lines, scale bars = 3 μm.

Our first report of RNG2 indicated that this protein associates with the apical complex early during cell division, whereas RNG1 is a late marker of daughter cell formation [28], [29]. To correlate RNG2 behavior with other known early events in daughter cell formation, we have used markers of the centrosome (centrin 1 antibodies) and centrocone (MORN1-cMyc expression) with the 3′ endogenous tagged RNG2-HA cells where RNG2 maintains its native promoter [29]. Duplication of the centrosome is one of the earliest events of daughter cell formation [37], and we see RNG2 association with daughter cells only after this event (Figure 3A–C). RNG2 first appears as two diffuse dots apparently in contact with each centrosome (Figure 3C). Subsequently, RNG2 resolves into a small ring that is separate and anterior to the centrosome (Figure 3D). This indicates a separation of these nascent structures, but we often see a small residual amount of RNG2 that persists with the centrosome (Figure 3D, arrowheads), suggesting a direct interaction with the centrosome occurs.

**Figure 4:**
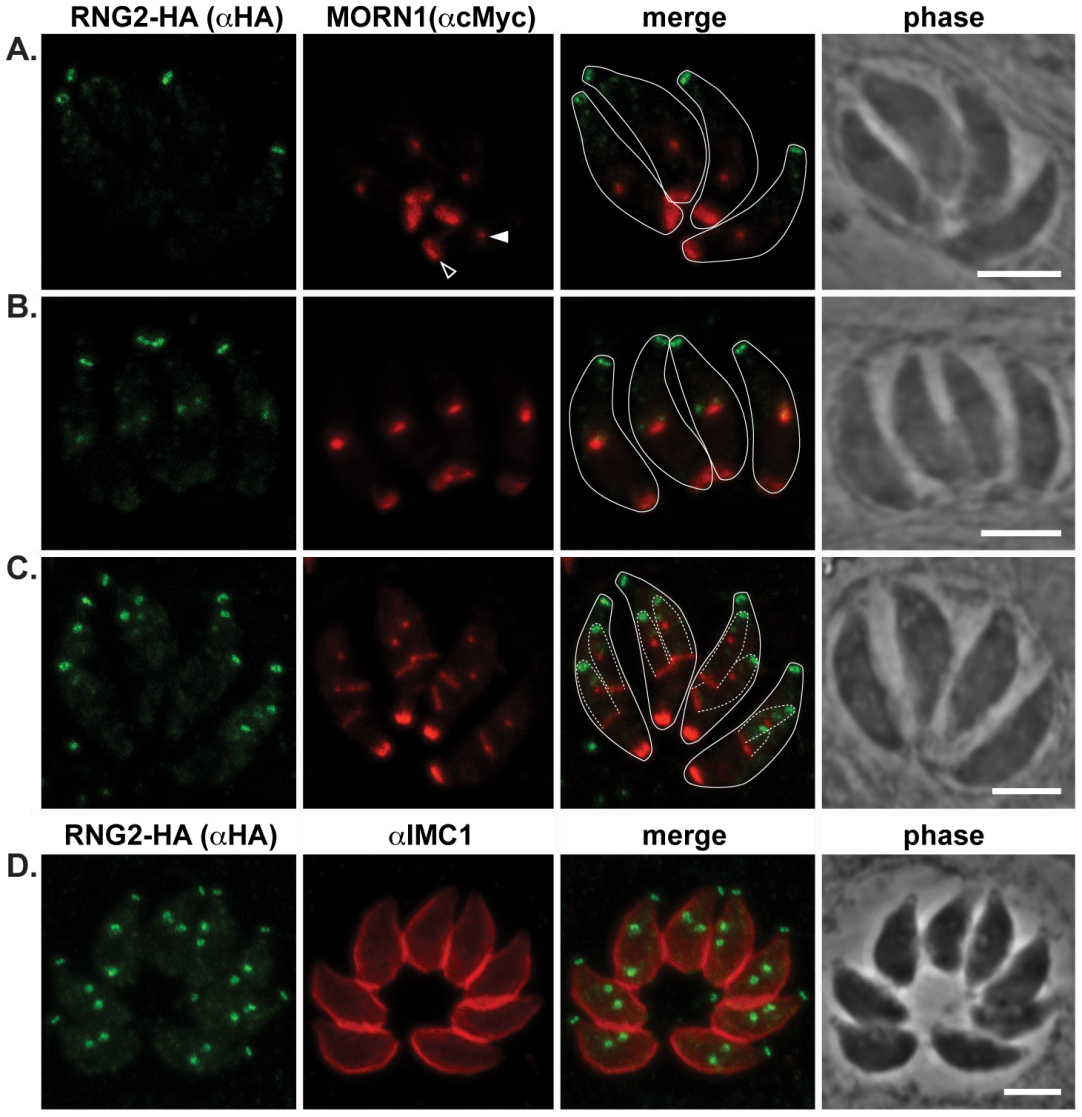
RNG2 appears before centrocone duplication or IMC1 association with daughter pellicles. (A, B, C) RNG2-HA (green) co-expressed with MORN1-cMyc (red). MORN1 can be seen at the basal complex of both mother (e.g. A, open arrowhead) and daughter cell pellicles, and at the centrocone (e.g. A, filled arrowhead). Inferred daughter buds shown with dashed lines (C) Elongation of the centrocone occurs prior to resolution of two centrocones and mitosis. (D) Immuno-labeled RNG2-HA (green) and IMC1 (red). Scale bars = 3 μm.

**Figure 5:**
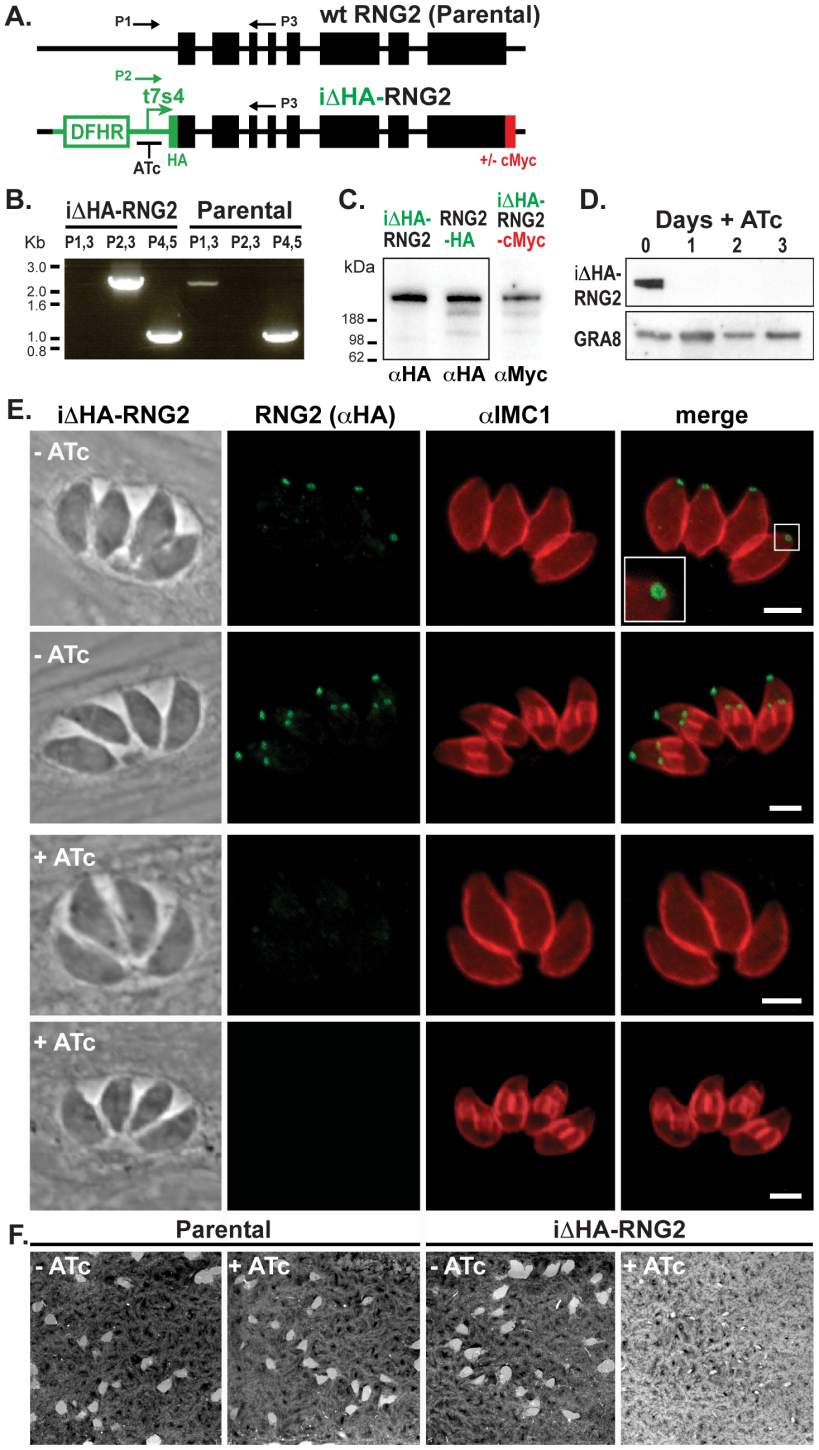
RNG2 is required for parasite growth. (A) Schematic of the chromosomal locus of wild type RNG2 and the insertional mutant iΔHA-RNG2 showing the tetracycline regulatable promoter (t7s4), N-terminal HA tag, C-terminal cMyc tag (integrated separately) selectable marker DHFR, exon structure of RNG2 (black boxes), and primers (P1–3) used to verify correct integration. (B) PCR analysis of genomic *rng2* locus after integration of the iΔHA insert. Primer P1–3 locations shown in (A) while P4,5 amplify *tic22* as a positive control. (C) Western blot of three versions of tagged RNG2 using either HA or cMyc antibodies (equal protein loading in all lanes). (D) Western blot of HA-RNG2 expression after 0 to 3 days of anhydrotetracycline (ATc) treatment. Dense granule protein GRA8 used as a loading control. (E) Immuno-fluorescence detection of HA-RNG2 (green) and cell pellicles (IMC1, red) after no or one day of ATc treatment. Scale bar = 3 μm. (F) Plaque assay measuring parasite growth over 8 days of either the parental or knockdown cell lines, in the absence (−) or presence (+) of ATc.

The centrocone is an elaboration of the nuclear envelope that serves as the connection point between centromeres of chromosomes in the nucleus to the extranuclear centrosome [38]. Following duplication of the centrosome and development of the mitotic spindle, the centrocone elongates before separating into two resolved centrocones. MORN1 labels the centrocone, as well as the basal complex of the IMC (seen as a ring at the base of the mother cell as well as forming daughter cells: Figure 4) [23], [39]–[41]. The nascent dots of RNG2 are seen before the centrocone separates into two structures, and before any MORN1 is associated with the IMC of the daughter buds (Figure 4B). RNG2 resolves into rings before the inner membrane complex protein IMC1 is associated with daughter cells (Figure 4D, see also Figure 5E). Together these data suggest that RNG2 is recruited to centrosomes immediately after their duplication, and then resolves into rings during initiation of daughter cell pellicle buds.

### RNG2 knockdown affects growth, but not cell replication or pellicle structure

To examine the function of RNG2 we generated an inducible knockdown cell line (iΔHA-RNG2) by 5′-replacement of the native RNG2 promoter with the tetracycline regulatable promoter (t7s4) in a Δku80/TATi background [42], [43]. An HA coding sequence was also appended to the 5′-terminus of the gene to follow RNG2 expression, and subsequently a cMyc tag at the 3-terminus (Figure 5A). Correct integration into the single *rng2* locus was verified by PCR, Western blot, and immunofluorescence microscopy. By PCR, the RNG2 coding region of the iΔHA-RNG2 mutant occurred downstream of the t7s4 promoter (P2,3; expected fragment size 2281 bp) and not the native promoter region (P1,3; expected fragment size 2134 bp) (Figure 5A and B). Western blots show that the N-terminal HA tag labels a >188 kDa protein in iΔHA-RNG2 cells, consistent with the predicted size of RNG2 (Figure 5C). This is of identical size to RNG2-HA that we previously generated by 3′ endogenous gene tagging (Figure 5C). Further, Western blotting against cMyc in the iΔHA-RNG2-cMyc cell line confirms this correct subsequent gene-tagging event, including some common minor presumed C-terminal processed or degradation products seen in both RNG2-HA and RNG2-cMyc cell lines (Figure 5C). Finally, immuno-localization of HA in iΔHA-RNG2 cells (and cMyc in iΔHA-RNG2-cMyc cells) (Figure 5E) shows the protein at apical rings that are first observed early in daughter cell formation, consistent with the RNG2-HA localization and behavior (Figures 1-4). Together, these data indicate that we have successfully replaced the native promoter of RNG2 with the regulatable t7s4 promoter.

The expression levels of HA-RNG2 in the iΔHA-RNG2 cells grown in the absence of the tetracycline analogue, anhydrotetracycline (ATc) is equivalent to RNG2-HA that is driven by the native promoter (Figure 5C). When iΔHA-RNG2 parasites were incubated in the presence of ATc (0.5 μg ml^−1^), HA-RNG2 expression was reduced to below detectable levels by Western blot within one day of ATc incubation (Figure 5D). Similarly, we were unable to detect HA-RNG2 protein by immunofluorescence assay (IFA) after one day of ATc incubation (Figure 5E). These data indicate effective knockdown of RNG2. To test for a growth phenotype associated with RNG2 knockdown, we measured growth across eight days using a plaque assay. In the absence of ATc, iΔHA-RNG2 cells developed plaques in host cell monolayers that were indistinguishable from plaques in the unmodified parental cell line (Figure 5F). When ATc was present, plaque sizes were dramatically reduced in iΔHA-RNG2 cells but not the parental cells, indicating that RNG2 plays a significant role in parasite proliferation.

**Figure 6:**
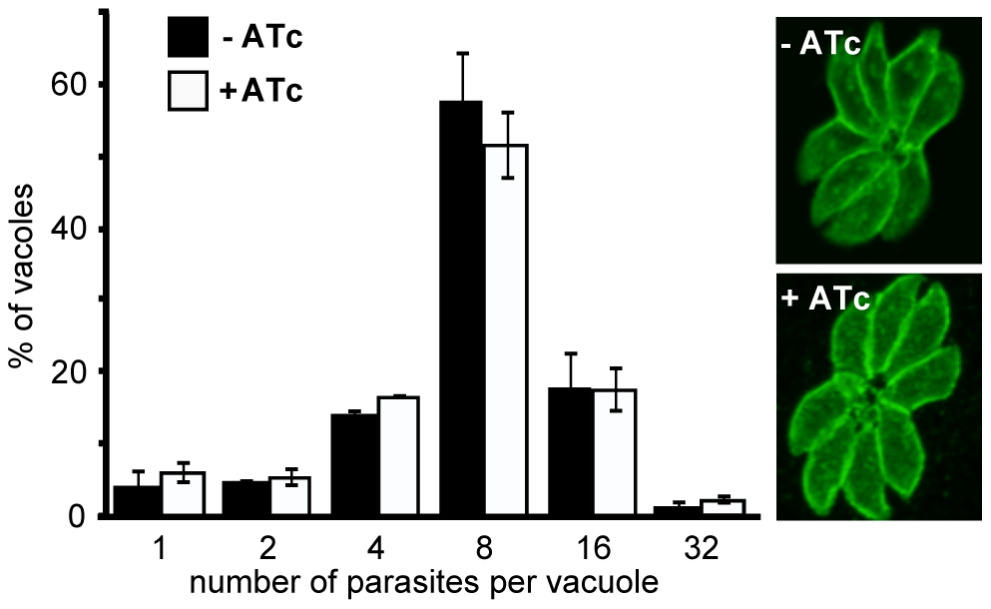
RNG2 is not required for intracellular parasite replication. Replication rate of iΔHA-RNG2 parasites grown with (+) or without (−) ATc treatment measured by parasite number per parasitophorous vacuole after 24 hours of growth post infection. >200 vacuoles were counted for each of three biological replicates, error bars = standard error of the mean. Representative SAG1-stained vacuoles show no gross difference between + or − ATc-treated cells.

The early presence of RNG2 during daughter pellicle bud assembly suggested that knockdown of RNG2 might perturb this important developmental stage and this could be responsible for the growth phenotype observed. We tested this by measuring parasite replication rates in host cells with or without RNG2 knocked down. iΔHA-RNG2 cells were cultured for three days, with or without ATc, then allowed to invade fresh host cells. After 24 hours of further incubation with or without ATc, infected monolayers were fixed and parasites immuno-stained with surface antigen SAG1 to count the number of parasites per vacuole. There was no significant difference in the replication rate of cells with or without RNG2 expression. Most vacuoles in both treatments contained eight cells (three division cycles), with equivalent distributions of vacuoles either lagging (two or four cells) or ahead (16 or 32 cells) of this replication state (Figure 6). The morphology of the +ATc replicating cells also appeared normal by SAG1 labeling, with the only notable difference being a reduction in the total number of vacuoles (see below).

**Figure 7:**
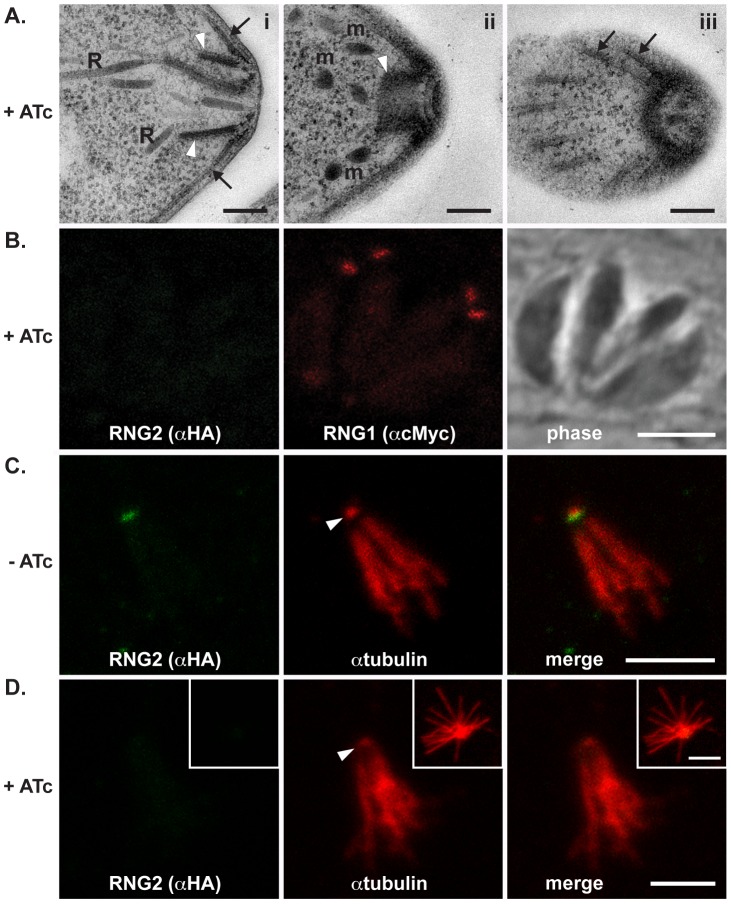
RNG2 knockdown results in no obvious structural change. (A) Transmission electron microscopy of ATc-treated parasites show typical apical structures. (i) Transverse section of apical complex showing rhoptries (R) extending within the conoid (white arrowheads) and microtubules and the IMC (arrows) converging on the apical polar ring. (ii) Oblique section of the apical complex shows the retracted conoid (arrowhead), preconoidal rings and micronemes (m), while a glancing section shows subpellicular microtubules (arrows) converging on the apical polar ring (iii).(B) iΔHA-RNG2 cells treated with ATc and co-expressing RNG1-cMyc show no change in RNG1 association with the apical polar ring. (C) Detergent-extracted pellicles show HA-RNG2 persists with the microtubular conoid (arrowhead) and sub-pellicular basket. (D) With ATc treatment and RNG2 knockdown these extracted pellicles remain unchanged with microtubules attached to the conoid (arrowhead). Inset image shows a splayed microtubular basket. Black scale bar = 200 nm, white scale bar = 3 μm.

We also tested for structural phenotypes associated with RNG2 knockdown. Formation of daughter inner membrane complexes, as measured by IFA, appeared unimpaired in parasites depleted of RNG2 (Figure 5E, bottom panel). Additionally, we examined the ultrastructure of cells grown with and without ATc by transmission electron microscopy and observed no differences in the apical complexes, including the apical polar ring, conoid, cell pellicle and position of rhoptries and micronemes (Figure 7A). The apical polar ring marker RNG1 was examined in cells replicating in the presence of ATc to determine if recruitment of this late ring marker requires RNG2. RNG1 localization to the apical polar ring was unchanged from wild type in the absence of RNG2 (Figure 7B), consistent with the loss of RNG2 having no effect on the structure of the apical polar ring. To test if the integrity of the sub-pellicular microtubular basket was perturbed by RNG2 knockdown, we performed detergent extraction of parasite pellicles. Without ATc, extracted pellicles were typical, with all microtubules anchored to the apical polar ring and conoid (Figure 7C). RNG2 rings remained stably associated with the extracted pellicles, indicating a detergent-resistant, strong association. Extracted pellicles from ATc-treated cells were identical with the exception of lacking RNG2 staining (Figure 7D). The ability of the conoid to be extruded from below the apical polar ring was also examined in knockdown cells. Extracellular parasites were stimulated using A23187 and the number of cells with everted conoids scored. No significant difference was seen in the rates of extrusion between iΔHA-RNG2 cells treated without ATc (81.4%, +/− 10.4% SD) or with ATc (84.6%, +/− 6.0% SD). We conclude that loss of RNG2 does not impair intracellular growth or daughter cell development.

### RNG2 knockdown perturbs invasion, motility and rhoptry evacuole formation

**Figure 8:**
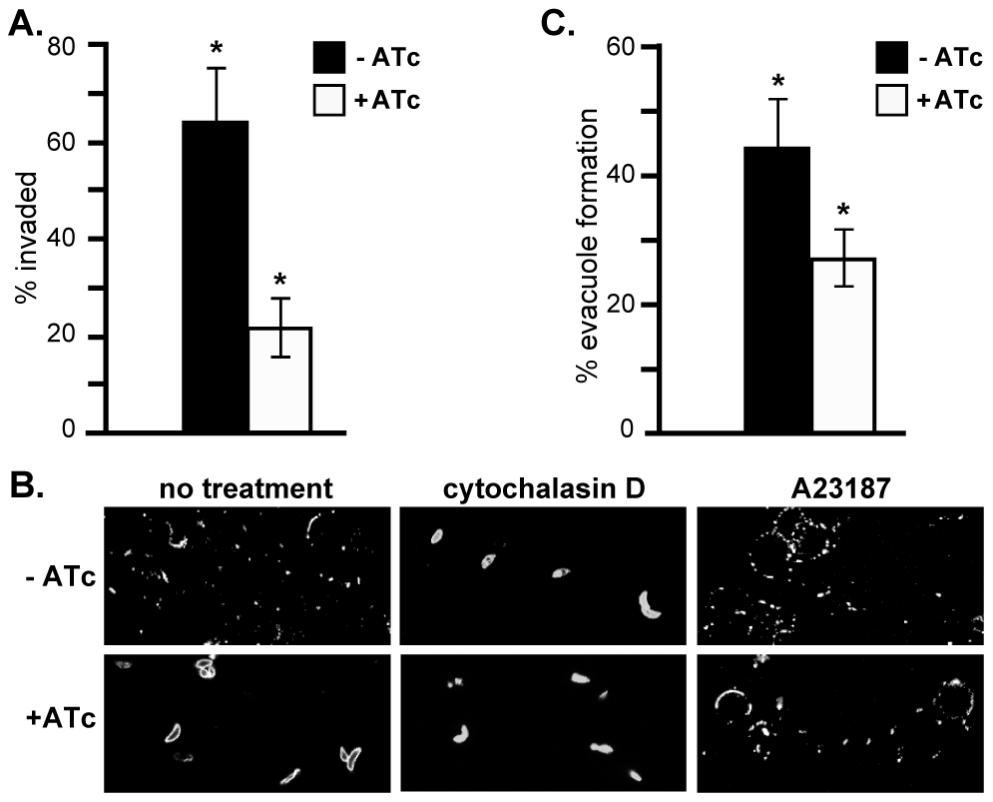
RNG2 is required for invasion, motility and rhoptry evacuole formation. (A) Percentage parasite invasion success of iΔHA-RNG2 cells grown either with or without ATc. >200 parasites were scored for each of three biological replicates. (B) Gliding motility detected by SAG1-positive trails of iΔHA-RNG2 cells with or without ATc. Motility was also assessed with further treatments of actin polymerization inhibitor cytochalasin D, or calcium ionophore A23187. (C) Percentage of ROP1-positive evacuoles formed by iΔHA-RNG2 cells grown with or without ATc. >200 parasites were scored for each of six biological replicates. Error bars  =  standard error of the mean, * denotes significant differences (P<0.05, two-tailed Student's t-test).

In the absence of a replication defect to explain the growth phenotype of RNG2 knockdown, we investigated host cell invasion. We assayed invasion efficiency using a red/green invasion assay [44], [45]. iΔHA-RNG2 parasites were cultured for two days with or without ATc, mechanically harvested from the host cells in potassium-rich Endo buffer, and then activated for invasion in low potassium buffer and allowed to invade host cells for 10 minutes. Invasion was then arrested by chemical fixation and parasites differentially immuno-stained according to whether they were outside or inside the host cells. The RNG2 knockdown parasites showed a strong invasion defect, with 66% reduction in invasion efficiency (Figure 8A).

Gliding motility is integral to invasion, and we tested the ability of iΔHA-RNG2 parasites to glide with or without ATc. Without ATc, parasites showed typical extracellular gliding activity, indicated by trails of surface protein SAG1 left on coated coverslips (Figure 8B). These trails were ablated by treatment with the actin inhibitor cytochalasin D. In RNG2 knocked down parasites (+ATc), we could see no such trails or evidence of gliding motility. Gliding, however, could be restored in RNG2 knockdown cells by treatment with the calcium ionophore A23187 (Figure 8B).

To further dissect the events of parasite invasion, we monitored evacuole formation, which is the release of rhoptry contents into the host cell [46]. To do this we allowed parasites to settle onto host cells in the presence of cytochalasin D. This enables parasites to apically attach to host cells, and secrete their rhoptry contents as evacuoles, but prevents them from further invasion. After 15 minutes, we fixed parasites and visualized evacuoles by immuno-staining with the rhoptry marker ROP1. Evacuoles can be seen as extended ROP1-positive emissions within the host cell. For cells grown without ATc, 44% of parasites formed evacuoles, which is consistent with controls in previous experiments (e.g. [47]). For iΔHA-RNG2 cells grown with ATc, only 27% of parasites generated evacuoles, which is a 39% reduction compared to controls (Figure 8C).

### RNG2 has a role in regulated secretion of micronemes

**Figure 9:**
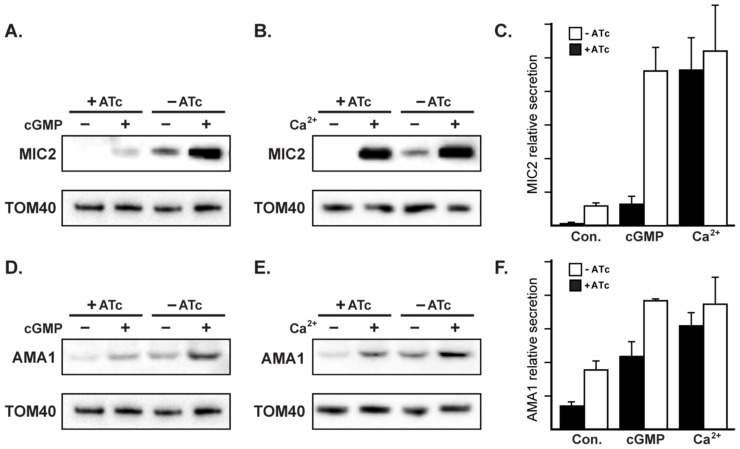
RNG2 has a role in regulated microneme secretion. Constitutive secretion of microneme proteins MIC2 (A, B) and AMA1 (D, E) from extracellular iΔHA-RNG2 cells grown with or without ATc. Secretion was also assessed with cGMP stimulation by Zaprinast (A, D), or with calcium stimulation by A23187 (B, E). Tom40 detection in cell pellets provides a control for parasite number used for secretion assays. (C, F) Secretion averages from biological replicates (n = 6 for constitutive (Con.) secretion; n = 4 for stimulated secretion). Error bars  =  standard error of the mean.

Staged release of invasion factors from secretory organelles—micronemes, rhoptries and dense granules—facilitates the coordinated events of parasite invasion and establishment of the parasitophorous vacuole [14]. In extracellular parasites, some secreted micronemal proteins translocate to the parasite surface, where they function in attachment of parasites to the host cells, a critical component of processes such as motility and invasion. Subsequent to their function, these micronemal proteins are cleaved by parasite proteases and released into the extracellular medium [48]. We assayed the release of two species of micronemal proteins, MIC2 and AMA1, from extracellular parasites. In the absence of ATc, iΔHA-RNG2 constitutively secreted both MIC2 and AMA1 (Figure 9). In ATc-treated iΔHA-RNG2 cells, we observed a marked microneme secretion defect for both MIC2 and AMA1, with little or no detectable protein in the extracellular medium (Figure 9). To check that MIC2 and AMA1 were synthesized to equivalent levels in ATc-treated cells we assayed for these proteins in intact parasites, and found no reduction in either microneme protein type (Figure S2A). IFAs against AMA1 and MIC2 similarly showed no difference in microneme distribution in the cells, predominantly towards the cell apex, when cells are depleted of RNG2 (Figure S2B, C). This indicates that the reduction in microneme proteins in the supernatant was caused by reduction in secretion rather than available protein.

Microneme secretion is regulated by two main signaling pathways: activation of calcium-dependent protein kinases (CDPKs) by calcium release from the ER; and activation of Protein Kinase G (PKG) by cGMP [49]–[51]. These two pathways can be experimentally manipulated by either inducing calcium store release into the cytoplasm with the ionophore A23187, or by elevating internal cGMP concentration by treatment with cGMP-specific phospho-diesterase inhibitor Zaprinast. Microneme secretion is upregulated by both A23187 and Zaprinast, and we saw equally robust secretion measured by MIC2 and AMA1 markers with both these stimuli in cells where RNG2 was present (-ATc) (Figure 9). In RNG2 knockdown parasites (+ATc) we also observed upregulation in microneme secretion (Figure 9). Interestingly, however, we observed distinct responses to calcium and cGMP stimuli. Calcium stimulation produced upregulation of secretion of both MIC2 and AMA1 in the knockdown cells to levels equivalent to the RNG2-expressing parasites (Figure 9C, F). Although cGMP stimulation did elevate MIC2 and AMA1 secretion, this was clearly less in RNG2 knockdown cells than when RNG2 was present (Figure 9C, F). To test this further, we stimulated microneme secretion with 8-Br-cGMP, an alternative activator of the cGMP pathway. We observed a similar muted response in microneme secretion when RNG2 was absent (Figure S3). These results suggest that RNG2 is critical for the constitutive secretion of micronemes, and demonstrates a relative insensitivity of microneme secretion to the cGMP pathway in parasites lacking RNG2.

## Discussion

The apical polar ring is the unifying structural feature of the apical complex, and arguably of all of Apicomplexa [10]. It provides a structural basis for the assembly of new daughter cells, and a focal point for invasion events into host cells. However, its molecular composition is poorly characterized, and its specific functions are experimentally untested. The *T. gondii* protein RNG2 provides insight into the early formation of this structure, its molecular interactions with other apical complex structures, and the molecular function of this gateway into the host cell.

The earliest events of cell division in *T. gondii* are extension and fission of the Golgi apparatus and the duplication of the centrosomes [20], [37], [52]. Immediately following centrosome duplication, the first recruitment of molecules associated with the new daughter cell pellicles is seen. Rab11B and IMC15 appear closely associated with the centrosomes shortly after their duplication [18], [19]. Rab11B is implicated in trafficking Golgi-derived vesicles to the developing alveolar sacs of the daughter cell pellicles, and IMC15 is one of the earliest pellicle scaffolding proteins that likely contributes to the coordination of alveolar sac assembly. We show that the earliest nascent components of the apical complex are also recruited to the centrosomes just after their division. RNG2 appears initially as a dot in contact with each centrosome before the mitotic spindle is evident. RNG2 then resolves into a ring associated with the apical polar ring as the nascent apical complex separates from its centrosome. Some residual RNG2 remains associated with the centrosome during daughter cell development suggesting that RNG2 interacts directly with the centrosome. Centrin2 is permanently associated with the centrosomes, but it also locates to the preconoidal rings, basal complex and peripheral annuli during daughter cell assembly [23], [25], [39]. It is unclear if centrosomal centrin2 contributes to these latter structures.

The apical polar ring acts as the microtubule organizing center that gives rise to the subpellicular array of 22 microtubules [8], [10]. Knockdown of RNG2 has no effect on daughter cell formation or stability of the microtubule array, so it is evidently neither necessary for ring formation or recruitment of other proteins required to form this ring. Rather, RNG2 is presumably itself recruited to the new ring. It therefore presents a conundrum as to why such early association of RNG2 within the apical complex occurs, if no early phenotype of its loss is evident. It is possible that the order of assembly of the apical complex might simply require an early recruitment of RNG2. The *T. gondii* pellicle is a remarkably robust and stable structure, including unusually static associated microtublues [11], and even after detergent extraction RNG2 remains in place. Perhaps correct integration of RNG2 in association with the apical polar ring and conoid necessitates early addition, despite the functional role awaiting daughter maturation.

A growing theme in apicomplexan biology is the key role of the centrosome in coordinating the essential structures of new daughter cells, including the Golgi apparatus, apicoplast, nucleus and pellicle buds [18], [37], [53]–[59]. Even after the daughter buds separate from the centrosomes, a striated fiber, homologous to rootlets of the flagellar apparatus found in flagellated cells, provides a tether between the centrosomes and the daughter pellicles as they continue to develop [57]. RNG2 suggests an additional, direct role for the centrosome in recruiting proteins that are then assembled into the new apical complexes. This behavior is similar to that seen in the basal bodies of ciliates that also appear to provide recruitment surfaces for repetitive, charged residue-containing proteins that are subsequently deployed to other cell pellicle structures [60]. Basal bodies are homologous structures to the centrioles of centrosomes, and RNG2 was identified by broad similarity to such pellicle proteins of ciliates [29].

**Figure 10:**
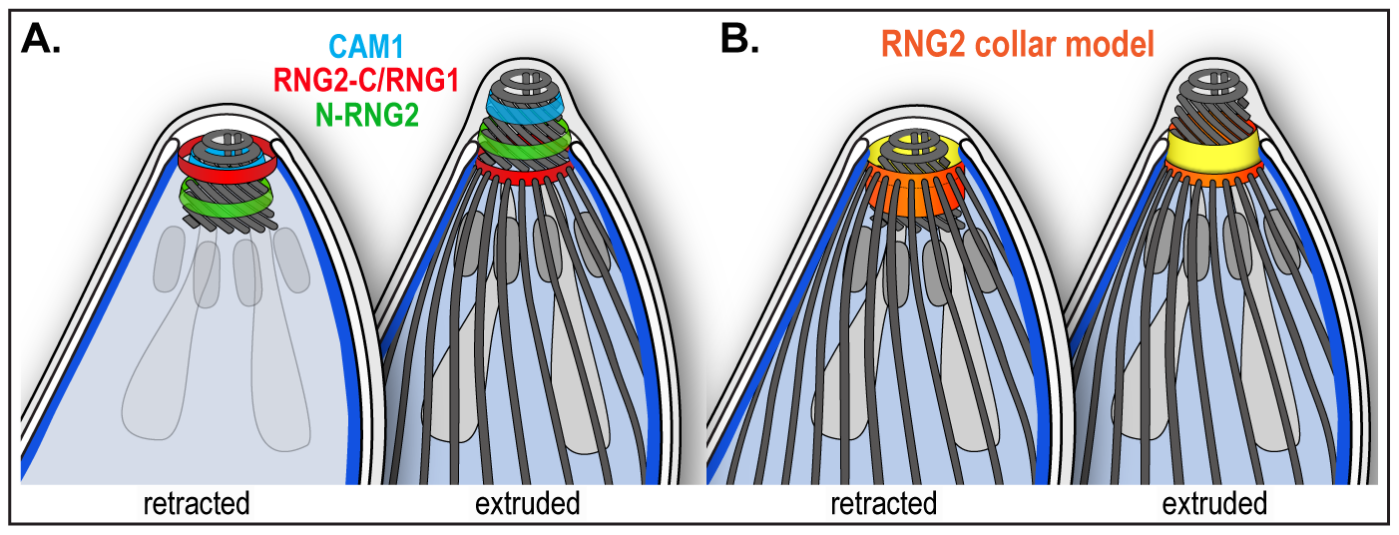
Schematic of RNG2 location within the apical complex. (A) Inferred positions of the N and C termini of RNG2 (labeled N-RNG2 and RNG2-C, respectively), conoid marker CAM1, and apical polar ring marker RNG1 within the structures of the apical complex, and with the conoid either retracted (subpellicular microtubules removed) or extruded. (B) Spatial model of RNG2 (orange and yellow), based on locations of protein termini, forming a collar between the apical polar ring and conoid. The collar is inverted upon conoid extrusion, potentially turning inside-out. See Figure 1 for full labeling of structures.

The resolvable displacement of the N- and C- termini of RNG2 in mature parasites, and their non-identical behavior during conoid extrusion, suggests that RNG2 interacts both with the apical polar ring and the conoid. The C-terminus forms a ring consistent with position, diameter and static behavior of the apical polar ring [8]. Predicted acylation sites (at least two palmitoylation sites by CSS-Palm 3.0, [61] using the highest stringency threshold, and up to eight) might facilitate association with the apical portion of the alveolar sacs, as is known to occur for some IMC-bound proteins [62]–[66]. The N-terminus, on the other hand, associates with the mobile conoid at a position posterior to the conoid protein CAM1. When the conoid is extruded the N-terminus is clearly anterior to the C-terminus, suggesting that it is not attached to the very base of the conoid which is aligned with the apical polar ring in this state [8]. The simplest model for RNG2, therefore, is that it forms a collar between the apical polar ring and the conoid (Figure 10). During conoid extrusion, a substantial reorientation of the RNG2 collar occurs, with the ring created by the N-terminus passing through that of the RNG2 C-terminus, and the collar turning inside-out (Figure 10B). It is notable that RNG2 knockdown does not effect conoid extrusion, indicating that RNG2 itself does not function in conoid extrusion. Instead, we speculate that conoid extrusion may mediate an altered RNG2 conformation, and changed presentation of RNG2 surfaces to other apical organelles or molecules. It is possible that this enables changed RNG2 function. Given the role of RNG2 in invasion—and more specifically in microneme secretion—conoid extrusion could function as an invasion-ready switch to enable RNG2 to perform its role.

The dramatic reduction in parasite growth with RNG2 knockdown provides the first experimental insight into the role of the apical polar ring in apicomplexan biology. Loss of RNG2 was associated with reduced parasite proliferation, motility and invasion. Secretion of both the micronemes and the rhoptries was markedly reduced. A reduction in microneme secretion alone could explain all of these phenotypes given that motility and evacuole formation are necessary for invasion, and are dependent on proteins secreted from micronemes such as the adhesin MIC2 for motility, MIC8 for rhoptry secretion and AMA1 for orientating the cell for moving junction formation [47], [67]–[70]. Reduction in microneme secretion was not due to any obvious perturbation of microneme formation or maturation as RNG2 depletion did not reduce microneme protein content or presentation of micronemes to the apical portion of the cells. The loss of microneme secretion in RNG2 knockdown cells was not absolute, with some secretion still occurring. Inhibition of secretion appeared to be more pronounced for MIC2 compared to AMA1. This might reflect different sensitivities of respective immuno-detections, or could indicate alternative secretion pathways for these two micronemal proteins. Recently, pools of different micronemal proteins have been shown to rely on distinct Rab-GTPases [71]. While both MIC2 and AMA1 were in the Rab5A/C-independent pools, it is unknown if there is further division amongst these different proteins.

Despite a reduction in invasion, approximately one third of RNG2 knockdown parasites were still able to invade, suggesting that they are able to overcome the microneme secretion defect. It is technically possible that, upon knockdown, there remains a residual amount of RNG2 protein that allows a low level of microneme secretion and subsequent invasion. Notably, however, recent knockout studies of the motility associated proteins MyosinA, MIC2 and Actin1 showed that parasites lacking these proteins were still able to invade host cells, albeit with dramatically reduced efficiency [72]. Similarly, approximately 15% of AMA1 knockdown cells remain invasion competent, suggesting this protein is also not completely essential to parasite invasion [47]. It is conceivable, therefore, that a RNG2-independent invasion pathway exists, mirroring the presumed “alternative” invasion pathways seen in the MyosinA, MIC2 and Actin1 knockouts [72].

The inhibition of microneme secretion by RNG2 knockdown was not due to a mechanistic block in secretion, as agonists of either the calcium- or cGMP-signaling pathways were able to reverse this inhibition. Indeed, the restoration of gliding motility in RNG2 knockdown cells by calcium stimulation (A23187) is consistent with this motility phenotype being due to lack of microneme secretion. Calcium and cGMP signaling have been implicated in regulation of key events in parasite invasion cycles [51], [73]–[75]. These molecules are believed to be second messengers for various extracellular stimuli, and in *T. gondii* act on up to twelve calcium-dependent protein kinases (CDPKs) and a cGMP-dependent protein kinase G (PKG), respectively [51]. While there is evidence of a division of labour amongst some of these different protein kinases for discrete events of invasion (e.g. TgCDPK1) and egress (e.g. TgCDPK1 and 3), there is also some level of redundancy and/or codependency on calcium and cGMP signaling [50], [64], [65]. For instance, while TgCDPK1 is required for microneme secretion during invasion [50], the PKG inhibitor compound 1 will also block this secretion [49].

We found that the response of microneme secretion in RNG2 knockdown cells to calcium stimulation was the same as that of cells retaining RNG2. However, for cGMP stimulation the response in the knockdowns was markedly less than in RNG2-expressing parasites (using either Zaprinast or 8-Br-cGMP). This indicates that parasites depleted in RNG2 are less sensitive to cGMP with respect to microneme secretion, and that RNG2 has some role either in cGMP sensing, or is downstream of PKG. If RNG2 has a role in the calcium signaling pathway then it is presumably upstream of calcium sensing, given that overriding calcium release provided full secretion. These data also indicate that activation of the calcium-dependent pathway can complement this cGMP pathway defect, whereas it cannot rescue a stronger inhibitor of the cGMP pathway such as compound 1 [49]. This suggests subtle interplay of multiple layers of regulation of these important cell processes, consistent with the observations of others [49], [64].

While the role of signaling molecules in the control of invasion events has been recognized for some time, this is the first insight into a location-specific function of the apical complex in these processes. RNG2 implicates the apical polar ring in control of microneme release, creating a regulated gateway for secretion at the apical complex. The precise site of microneme secretion is controversial, with some arguing that release occurs between the apical polar ring and the base of the extruded conoid [76], rather than at the apical aperture of the conoid. The location of RNG2 between the apical polar ring and conoid base is therefore intriguing, however, we cannot currently say whether this supports basal release, or whether RNG2 controls the onward traffic of micronemes to the conoid apex. RNG2 activity in these processes may be activated directly by CDPK and/or PKG phosphorylation, and phosphoproteomic studies have identified numerous phosphorylated sites in RNG2, including one calcium-dependent event [30], [77], [78]. Alternatively, it is possible that RNG2 may recruit other proteins to the apical polar ring that provide a link in these phospho-signaling events. The repetitive and high charged amino acid content of the class of proteins to which RNG2 belong [29], and prediction of coiled-coil domains, is consistent with facilitating protein-protein interactions.

The sequence of RNG2 is apparently fast evolving, with the homologue in closely related *Neospora caninum* sharing only 58% amino acid identity. Consequently, homologues are difficult to identify in more distantly related apicomplexans. Nevertheless, we predict that the function of secretion regulation that RNG2 confers on the polar ring of the apical complex is likely conserved throughout Apicomplexa. Possible homologues of the apical complex are also evident in the early ancestor lineages of apicomplexans (Chromerids and Colpodelids) as well as some some early-diverging members of the neighboring dinoflagellate lineage (Perkinsids and Psammosa) [79]–[83]. These organisms include symbionts, micropredators and parasites, and are believed to use their apical complex for myzocytoic feeding or entry into their metazoan partners. In all these lineages, conspicuous putative secretory organelles are clustered around structural elements of an apical complex, which are intimately associated with, and perhaps derived from, the flagellar apparatus of these flagellate organisms. Recent reports of flagellar-associated proteins (striated fiber assemblins and SAS6L) contributing to *T. gondii* apical complex assembly corroborate this ancestral state of the apical complex [24], [57]. Moreover, the cGMP signaling pathway is known to correlate specifically with presence of flagella, where it contributes to the important flagellum function of environmental sensing and signal transduction [84]. Together these observations suggest that a key feature of the evolution of the apical complex was likely specialization of flagellar-associated structures in the regulated delivery of secreted factors that facilitate predation and ultimately parasitism.

## Materials and Methods

### Growth and generation of protein-tagged and knockdown parasites

*T. gondii* tachyzoites were grown by serial passage in human foreskin fibroblast (HFF) cells as previously described [85]. RNG2-HA parasites were previously generated by 3′ endogenous tagging with 3XHA coding sequence of the *rng2* gene (toxodb.org gene ID:TgME49_244470) [29]. To generate the conditional RNG2 knockdown parasite strain (iΔHA-RNG2), we began by amplifying 2015 bp upstream of the RNG2 start codon (5′ flank) using the primers 5′-CTGACATATGGAGACTGCCACAAAGGAAGGTACAC and 5′-GATCATCCATCGAAACGCTCCGTGACGGAAGTA. We digested the product with *Nsi*I and *Nde*I and ligated this into the equivalent sites of the vector pPR2-HA3 (Chris Tonkin and GvD, unpublished), a modified version of the vector pPR (a kind gift from Lilach Sheiner, U. Georgia; [42]). We next amplified a 2042 bp fragment beginning at the start codon of RNG2 (3′ flank) with the primers 5′-GATCCCCGGGATGCACCCCCACCTTTCTTCCGCAG and 5′-CGATGCGGCCGCGACGGTGGTGTTATTGATTGGTTGC. We digested this with *Xma*I and *Not*I and ligated this into equivalent sites of the pPR2-HA3 vector that already contained the RNG2 5′ flank. The resulting vector positions the first RNG2 codon downstream of the ATc-regulatable t7s4 promoter and a 3xHA tag. We linearized the resulting vector with *Not*I and transfected this into TATiΔku80 parasites (a kind gift from Lilach Sheiner and Boris Striepen, U. Georgia; [42]). Parasites were selected with pyrimethamine and cloned by limiting dilution. To identify parasite clones where the t7s4 promoter had successfully replaced the native RNG2 promoter, we utilized the primers P1 (5′- CAGATTCCGAATTCTTTGG), P2 (5′-TGTAGAGCTGGTGCGTGAG) and P3 (5′-AAGGGGACGCAGTTCTCGGA) in the combinations described in Figure 5A. For RNG2 cMyc tagging, we PCR amplified a 3′ fragment of *rng2* gene using the primers 5′- GATCAGATCTGCAGCTGACACACTCCTGACG and 5′- GCATTCTAGAGTTTGTTGATGCGTCCGAGACAAC, digested this with *Bgl*II and *Xba*I and ligated into the *Bgl*II and *Avr*II sites of the vector pgCM3, a vector that fuses the 3′ region of a gene-of-interest with a 3× cmyc tag (NK and GvD, unpublished). This vector was linearized with *Avr*II, transfected into the iΔHA-RNG2 strain, selected on chloramphenicol and cloned by limiting dilution. RNG2 knockdown was induced by culturing with 0.5 μg ml ^−1^ of anhydrotetracycline (ATc). All PCRs were performed with Phusion polymerase (Thermo Scientific).

To tag the C-terminus of RNG2 with GFP, we digested the pCTG vector [86] with *Avr*II and *Bam*HI and ligated this into the equivalent sites of the pHA_3_-LIC-DHFR(RNG2) vector [29]. This exchanged the 3xHA tag with a GFP tag in a RNG2 3′ replacement vector that we termed pGFP-LIC-DHFR(RNG2). We linearized the resultant vector with *Nsi*I, transfected this into the TATi/ΔKu80 parasites [42] and selected on pyrimethamine. We cloned the resulting drug resistant parasites and confirmed expression of RNG2-GFP by microscopy. We next tagged the N-terminus of RNG2-GFP with a mCherry-3× c-myc tag through a promoter replacement strategy. First, we digested the pPR2-HA_3_(RNG2) vector described above with *Nhe*I and *Xma*I to excise the 3x-HA tag. We then digested the mCherry-3× c-myc tag from the vector pCTChM3 (a kind gift from Chris Tonkin, Walter and Eliza Hall Institute) with *Nhe*I and *Xma*I and ligated the resulting fragment into the pPR2 (RNG2) vector to generate the vector we termed pPR2-GFP(RNG2). We next replaced the pyrimethamine-resistance cassette in this vector with a chloramphenicol-resistance cassette. We PCR amplified the chloramphenicol-resistance cassette from the vector pgCM_3_ using the primers 5′-GATCATGCATAAAACCCTCGAAGGCTGCTAGTAC and 5′-GATCACTAGTGGATCCCCCTCGGG. The resulting PCR product was digested with *Spe*I and *Nsi*I and ligated into the equivalent sites of the pPR2-GFP(RNG2) vector to generate a vector we termed pPR2-CAT-GFP(RNG2). We linearized this vector with *Not*I and transfected this into the RNG2-GFP cell line, selecting on chloramphenicol. We cloned drug-resistant parasites, and confirmed integration through Western blotting.

For C-terminal 3XcMyc-tagged RNG1 and CAM1 (via 3′ endogenous gene replacement) the coding sequence of each gene was amplified and cloned into pBTM3 (GvD, unpublished). The primers used to amplify the coding sequence of RNG1 were 5′- GATCAGATCTAAAATGGCGCTAATTCCCTCGC and 5′- GATCCCTAGGCGCCAGGTAGTAGACAGGTGGA, and CAM1 were 5′-GATCCCTAGGTTTATTCGCGGAAGGCAGAGAC and 5′-TGGACTGTGGTCGACGCAGAAG. For transient expression of RNG1-GFP, the 3XcMyc tag was removed from the RNG1-cMyc vector by digestion with *Avr*II and *Not*1 and GFP coding sequence ligated in its place. The CAM1-GFP vector was a kind gift by Martin Blume, Bio21, Australia. To label MORN1 we transiently transfected parasites with a cMyc-tagged MORN1 plasmid (a kind gift from MJ Gubbels, Boston College).

### Microscopy

Immunofluorescence assays (IFAs) were performed as previously described [87] using antibodies and their concentrations listed in Supplemental Table S1. 3D-SIM was implemented on a DeltaVision OMX V4 Blaze (Applied Precision) with samples prepared as described [88], excited using 488 and 568 nm lasers and imaged using band pass filters at 528 and 608 with a 60× oil immersion lens (1.42 NA). Parasite pellicles were extracted in deoxycholate as previously described [66]. Briefly, we filtered parasites through a 3 μm filter and resuspended them in phosphate-buffered saline. We attached parasites to coverslips with 0.1% polyethyleneimine (PEI) and extracted in 10 mM deoxycholate for 10 min at room temperature. We fixed parasites in 4% paraformaldehyde for 10 min and then proceeded as for IFAs. Cells or cell extracts were analyzed on a Leica TCS SP2 confocal laser-scanning microscope. Only the brightness/contrast ratio of the images was modified, using Adobe Photoshop CS4. For live cell imaging, parasites were incubated in glass-bottomed dishes (MatTek) in phenol-red free Dulbecco's modified Eagle's medium supplemented with 1% foetal calf serum and antibiotics. During imaging, parasites were incubated in a 5% CO_2_/air atmosphere in a humidified 37°C chamber. Imaging was performed using a DeltaVision set-up with an inverted Olympus IX71 microscope, an Olympus objective lens (UPlanSApo, 100×/1.40 oil), and a Photometrics CoolSNAP HQ^2^ camera. Images were acquired using 2×2 binning, and deconvolved prior to linear adjustment of contrast and brightness. For transmission electron microscopy, parasites were cultured for three days on 0.5 μg ml^−1^ ATc, fixed in PBS with 2.5% paraformaldehyde and 1% glutaraldehyde, post fixed in 1% OsO_4_, and pellet-embedded in 1% low-melting agarose. The agarose block was ethanol dehydrated, embedded in LR White resin and polymerized. Ultrathin sections were cut on a Leica Ultracut R microtome, lead and uranium stained and visualized with a Philips CM120 BioTWIN transmission electron microscope at 120 kV.

### Western blot analysis

Antibodies and their concentrations used for Western blots are listed in Table S1. Parasites were filtered through a 3 μm filter, counted by haemocytometer and solubilized in sample buffer (Invitrogen) at equivalent cell densities. Standard Western blot detection was performed, with Horse Radish Peroxidase conjugated secondary antibodies detected using SuperSignal West Pico Chemiluminescent Substrate (Pierce). Signal strength was quantified using a BioRad Chemidoc imager.

### Growth, replication and conoid extrusion assays

For growth assays extracellular parasites were filtered, counted by haemocytometer, and 500 parasites added to 25 cm^2^ tissue culture flasks containing a confluent monolayer of HFF cells. ATc (0.5 μg ml^−1^) was added from the outset of the experiment. To visualize plaque sizes, flasks were aspirated, fixed with 5 ml 100% ethanol (5 minutes), stained with 5 ml crystal violet solution (15 minutes) then washed once with 1× phosphate-buffered saline (PBS) and dried before imaging. For replication assays, parasites grown for three days with or without ATc (0.5 μg ml^−1^), harvested and filtered. Equal numbers were allowed to invade HFF cells on coverslips for two hours. Coverslips were washed three times with Dulbecco's modified Eagle's medium (DMEM) (supplemented with 1% FCS, 0.2 mM L-glutamine) to remove uninvaded parasites, and cultured for 24 hours with ongoing +/− ATc regimens. Cells were then fixed and processed for SAG1 IFA and parasite number per parasitophorous vacuole scored. To assess conoid extrusion ability, parasites were grown for three days with or without ATc, harvested, filtered and resuspened in DMEM to 2.5×10^7^ parasites ml^−1^. A23187 was added to samples at a final concentration of 5 μM (or equivalent volume of DMSO as a control), and parasites incubated for 30 seconds at 37 °C, then fixed with 1.25% glutaraldehyde and settled on PEI coated coverslips. Conoid extrusion was scored by phase microscopy with >200 cells counted per replicate (n = 3).

### Invasion, motility and evacuole assays

Red/green invasion assays were performed as described previously [44], [45]. Briefly, parasites were grown for two days with or without ATc, harvested within HFF cells by trypsinisation, then mechanically released by passage through a 26 gauge needle in Endo Buffer (44.7 mM K_2_SO_4_, 10 mM MgSO_4_, 106 mM sucrose, 5 mM glucose, 20 mM Tris-H_2_SO_4_, 3.5 mg/ml BSA, pH 8.2). Cells were counted and resuspended to 2.5×10^7^ parasites ml^−1^, and 200 μl allowed to settle onto HFF cells on coverslips in Endo buffer for 20 minutes. Endo Buffer was then aspirated, and replaced with 200 μl of Invasion Buffer (DMEM supplemented with 3% FCS, 10 mM HEPES, pH 7.4). After 10 minutes at 37 °C, cells were fixed with 2.5% Paraformaldehyde and 0.02% glutaraldehyde in PBS, blocked, then probed with anti-SAG1 (Abcam) to label uninvaded cells. Samples were then permeabilized (0.25%TX100 in 1xPBS) for 10 minutes and probed with anti-GAP45 to label all cells. IFAs were completed with secondary antibodies as normal, and then imaged using a Leica SP2 confocal microscope. Fields of view were selected observing the green (anti-GAP45) channel only to eliminate biased selection of parasites. Images were processed using the Leica SP2 software, and labeled cells scored according to invaded (red) or uninvaded (red and green). A minimum of 200 parasites were scored for each of three biological replicates, and invasion percentage calculated as invaded over total parasites.

For motility assays, PEI coated coverslips were incubated with fetal calf serum for two hours. Parasite cultures grown for two days with or without ATc were needle passed, filtered and resuspended to 10^7^ cells ml^−1^. 1 ml of parasites was placed on coverslip with either no drug (plus DMSO to equivalent of drug samples), 1 μm cytochalasin D, or 5 μM A23187, and incubated at 37 °C for 90 minutes. Samples were then fixed and SAG1 IFAs performed. Evacuole assays were performed as previously described [47].

### Secretion assay

Parasite cultures grown for two days with or without ATc were harvested, pelleted, washed with Invasion Buffer and resuspended to 2.5×10^8^ cells ml^−1^. Cells were maintained at 20°C in all steps post harvesting. 50 μl of parasite suspensions were mixed separately with an equal volume of either Invasion Buffer alone (plus DMSO to equivalent of drug samples), 1.0 mM μM Zaprinast, 10 μM A23187 or 10 mM 8-Br-cGMP (final concentrations 0.5 mM, 5 μm, and 5 mM, respectively). Cells were incubated at 37°C for 20 minutes to allow secretion, then arrested on ice for 2 minutes before parasites were separated from secreted soluble proteins by centrifugation at 8000rpm at 4°C for 2 minutes. 85 μl of supernatant was removed, centrifuged at 8000rpm at 4°C for 2 minutes to remove any remaining cells, and 75 μl removed and boiled with Sample Buffer as the secreted protein fraction. The pelleted cells were washed with PBS and then boiled with Sample Buffer. Secreted protein samples were analyzed for MIC2 and AMA1 by Western blot, and cell pellets analyzed for Tom40 to verify equal cell numbers used for the different assay conditions.

## Supporting Information

Figure S1 **Live cell imaging of the N- and C-termini of RNG2.** (A-B) A Western blot depicting mCherry-cMyc-RNG2-GFP parasites probed with (A) anti-cMyc and (B) anti-GFP. In both blots, the masses of the tagged RNG2 protein are equivalent in size (>260 kDa), indicative of successful targeting of both termini of the gene. (C) Live cell imaging of mCherry-cMyc-RNG2-GFP intracellular parasites. Arrowheads depict the apical ring. In all parasites, N-terminal mCherry labeling is posterior to the C-terminal GFP labeling. (i) In the newly formed apical rings of daughter buds, the N-terminus of RNG2 is also posterior to the C-terminus (bottom panel, arrows). Note: fluorescence is also retained in the residual bodies. (D) Live cell imaging of mCherry-c-myc-RNG2-GFP parasites in extracellular parasites. (i) N-terminal mCherry labeling is posterior to the C-terminal GFP-labeling when the conoid is retracted. (ii) Treatment with Ca^2+^ ionophore A23187 causes conoid extrusion, and relocation of the N-terminus of RNG2 to the anterior side of the C-terminus. Scale bars are 2 μm.
(PDF)

Figure S2 **Western blot and immunofluorescence assays for microneme maturity with RNG2 depletion.** (A) In parasite total protein samples, microneme proteins MIC2 and AMA1, and mitochondrial protein Tom40, show equivalent amounts of protein in iΔHA-RNG2 cells treated with or without ATc for three days. Only HA-RNG2, detected by HA antibodies, shows depletion with ATc treatment. Equal cell numbers were used in all gel lanes. (B, C) IFA detection of (B) AMA1 and HA-RNG2, or (C) MIC2 in intracellular iΔHA-RNG2 cells treated with or without ATc for two days. Scale bar = 5 μm.
(PDF)

Figure S3 **8-Br-cGMP-stimulation of microneme secretion is muted in RNG2 minus cells.** MIC2 secretion without RNG2 (iΔHA-RNG2 cells +ATc) or with RNG2 (iΔHA-RNG2 cells -ATc and parental cells). Constitutive MIC2 secretion, and secretion with exogenous cGMP (by analogue 8-Br-cGMP) (A), or calcium stimulation (by ionophore A23187) (B) is assayed by Western blot. Stimulated microneme secretion by exogenous calcium is strong in all cells, but by exogenous cGMP is reduced in RNG2 knockdown cells.
(PDF)

Table S1 **Antibodies used for microscopy and protein assays.**
(PDF)

## References

[1] Adl SM, Leander BS, Simpson AGB, Archibald JM, Andersen OR, et al (2007) Diversity, Nomenclature, and Taxonomy of Protists. Systematic Biol 56: 684–689. 10.1080/10635150701494127 17661235

[2] WHO (2012) World Malaria Report 2012.: Geneva, Switzerland: World Health Organisation.

[3] Montoya JG, Liesenfeld O (2004) Toxoplasmosis. Lancet 363: 1965–1976. 15194258 10.1016/S0140-6736(04)16412-X

[4] Leander BS (2008) Marine gregarines: evolutionary prelude to the apicomplexan radiation? Trends Parasitol 24: 60–67. 18226585 10.1016/j.pt.2007.11.005

[5] Simdyanov TG, Kuvardina ON (2007) Fine structure and putative feeding mechanism of the archigregarine Selenidium orientale (Apicomplexa: Gregarinomorpha). Eur J Protistol 43: 17–25. 17126539 10.1016/j.ejop.2006.09.003

[6] Gubbels M-J, Duraisingh MT (2012) Evolution of apicomplexan secretory organelles. Int J Parasitol 42: 1071–1081. 23068912 10.1016/j.ijpara.2012.09.009 PMC3583008

[7] Baum J, Gilberger T, Frischknecht F, Meissner M (2008) Host-cell invasion by malaria parasites: insights from *Plasmodium* and *Toxoplasma* . Trends Parasitol 24: 557–563. 18835222 10.1016/j.pt.2008.08.006

[8] Nichols BA, Chiappino ML (1987) Cytoskeleton of *Toxoplasma gondii* . J Protozool 34: 217–226. 3585817 10.1111/j.1550-7408.1987.tb03162.x

[9] Russell DG, Burns RG (1984) The polar ring of coccidian sporozoites: a unique microtubule-organizing centre. J Cell Sci 65: 193–207. 6715423 10.1242/jcs.65.1.193

[10] Morrissette NS, Sibley LD (2002) Cytoskeleton of apicomplexan parasites. Microbiol Mol Biol Rev 66: 21–38. 11875126 10.1128/MMBR.66.1.21-38.2002 PMC120781

[11] Morrissette NS, Murray JM, Roos DS (1997) Subpellicular microtubules associate with an intramembranous particle lattice in the protozoan parasite *Toxoplasma gondii* . J Cell Sci 110: 35–42. 9010782 10.1242/jcs.110.1.35

[12] Anderson-White BR, Ivey FD, Cheng K, Szatanek T, Lorestani A, et al (2011) A family of intermediate filament-like proteins is sequentially assembled into the cytoskeleton of *Toxoplasma gondii* . Cell Microbiol 13: 18–31. 20698859 10.1111/j.1462-5822.2010.01514.x PMC3005026

[13] Hu K, Roos DS, Murray JM (2002) A novel polymer of tubulin forms the conoid of *Toxoplasma gondii* . J Cell Biol 156: 1039–1050. 11901169 10.1083/jcb.200112086 PMC2173456

[14] Carruthers VB, Sibley LD (1997) Sequential protein secretion from three distinct organelles of *Toxoplasma gondii* accompanies invasion of human fibroblasts. Eur J Cell Biol 73: 114–123. 9208224 PMC13475723

[15] Brockley Paterson W, Desser SS (1989) The polar ring complex in ookinetes of Leucocytozoon simondi (Apicomplexa: Haemosporina) and evidence for a conoid in haemosporidian ookinetes. Eur J Protistol 24: 244–251. 23195660 10.1016/S0932-4739(89)80061-6

[16] Aikawa M (1967) Ultrastructure of the pellicular complex of Plasmodium fallax. J Cell Biol 35: 103–113. 6061711 10.1083/jcb.35.1.103 PMC2107124

[17] Sheffield HG, Melton ML (1968) The fine structure and reproduction of *Toxoplasma gondii* . J Parasitol 54: 209–226. 5647101

[18] Anderson-White B, Beck JR, Chen C-T, Meissner M, Bradley PJ, et al (2012) Cytoskeleton Assembly in *Toxoplasma gondii* Cell Division. In: Jeong KS, editor. International Review Of Cell and Molecular Biology. Burlington: Academic Press, Vol. 298 . pp. 1–31. 10.1016/B978-0-12-394309-5.00001-8 PMC4066374 22878103

[19] Agop-Nersesian C, Egarter S, Langsley G, Foth BJ, Ferguson DJP, et al (2010) Biogenesis of the inner membrane complex is dependent on vesicular transport by the alveolate specific GTPase Rab11B. PLOS Pathog 6: e1001029. 20686666 10.1371/journal.ppat.1001029 PMC2912401

[20] Hu K, Mann T, Striepen B, Beckers CJM, Roos DS, et al (2002) Daughter cell assembly in the protozoan parasite *Toxoplasma gondii* . Mol Biol Cell 13: 593–606. 11854415 10.1091/mbc.01-06-0309 PMC65652

[21] Kono M, Herrmann S, Loughran NB, Cabrera A, Engelberg K, et al (2012) Evolution and architecture of the inner membrane complex in asexual and sexual stages of the malaria parasite. Mol Biol Evol 29: 2113–2132. 22389454 10.1093/molbev/mss081

[22] Striepen B, Jordan CN, Reiff S, van Dooren GG (2007) Building the perfect parasite: cell division in apicomplexa. PLOS Pathog 3: e78. 17604449 10.1371/journal.ppat.0030078 PMC1904476

[23] Hu K, Johnson J, Florens L, Fraunholz M, Suravajjala S, et al (2006) Cytoskeletal components of an invasion machine–the apical complex of *Toxoplasma gondii* . PLOS Pathog 2: e13. 16518471 10.1371/journal.ppat.0020013 PMC1383488

[24] de Leon JC, Scheumann N, Beatty W, Beck JR, Tran JQ, et al (2013) A SAS-6-like protein suggests that the *Toxoplasma* conoid complex evolved from flagellar components. Euk Cell 12: 1009–1019. 10.1128/EC.00096-13 PMC3697468 23687115

[25] Liu J, Wetzel L, Zhang Y, Nagayasu E, Ems-McClung S, et al (2013) Novel thioredoxin-like proteins are components of a protein complex coating the cortical microtubules of *Toxoplasma gondii* . Euk Cell 12: 1588–1599. 10.1128/EC.00082-13 PMC3889574 23873863

[26] Heaslip AT, Ems-Mcclung SC, Hu K (2009) TgICMAP1 Is a novel microtubule binding protein in *Toxoplasma gondii* . PLOS ONE 4: e7406. 19823689 10.1371/journal.pone.0007406 PMC2758671

[27] Carey KL, Westwood NJ, Mitchison TJ, Ward GE (2004) A small-molecule approach to studying invasive mechanisms of *Toxoplasma gondii* . Proc Natl Acad Sci USA 101: 7433–7438. 15123807 10.1073/pnas.0307769101 PMC409936

[28] Tran JQ, De Leon JC, Li C, Huynh M-H, Beatty W, et al (2010) RNG1 is a late marker of the apical polar ring in *Toxoplasma gondii* . Cytoskeleton 67: 586–598. 20658557 10.1002/cm.20469 PMC2998517

[29] Gould SB, Kraft LGK, van Dooren GG, Goodman CD, Ford KL, et al (2011) Ciliate pellicular proteome identifies novel protein families with characteristic repeat motifs that are common to alveolates. Mol Biol Evol 28: 1319–1331. 21127172 10.1093/molbev/msq321

[30] Gajria B, Bahl A, Brestelli J, Dommer J, Fischer S, et al (2008) ToxoDB: an integrated *Toxoplasma gondii* database resource. Nucleic Acids Res 36: D553–D556. 18003657 10.1093/nar/gkm981 PMC2238934

[31] Behnke MS, Wootton JC, Lehmann MM, Radke JB, Lucas O, et al (2010) Coordinated progression through two subtranscriptomes underlies the tachyzoite cycle of *Toxoplasma gondii* . PLOS ONE 5: e12354. 20865045 10.1371/journal.pone.0012354 PMC2928733

[32] Lupas A, Van Dyke M, Stock J (1991) Predicting coiled coils from protein sequences. Science 252: 1162–1164. 2031185 10.1126/science.252.5009.1162

[33] Hanssen E, Carlton P, Deed S, Klonis N, Sedat J, et al (2010) Whole cell imaging reveals novel modular features of the exomembrane system of the malaria parasite, Plasmodium falciparum. Int J Parasitol 40: 123–134. 19766648 10.1016/j.ijpara.2009.09.004

[34] Schermelleh L, Carlton PM, Haase S, Shao L, Winoto L, et al (2008) Subdiffraction multicolor imaging of the nuclear periphery with 3D structured illumination microscopy. Science 320: 1332–1336. 18535242 10.1126/science.1156947 PMC2916659

[35] Mondragón R, Frixione E (1996) Ca(2+)-dependence of conoid extrusion in *Toxoplasma gondii* tachyzoites. J Eukaryot Microbiol 43: 120–127. 8720941 10.1111/j.1550-7408.1996.tb04491.x

[36] Del Carmen MG, Mondragón M, González S, Mondragón R (2009) Induction and regulation of conoid extrusion in *Toxoplasma gondii* . Cell Microbiol 11: 967–982. 19416276 10.1111/j.1462-5822.2009.01304.x

[37] Hartmann J, Hu K, He CY, Pelletier L, Roos DS, et al (2006) Golgi and centrosome cycles in *Toxoplasma gondii* . Mol Biochem Parasitol 145: 125–127. 16266757 10.1016/j.molbiopara.2005.09.015

[38] Brooks CF, Francia ME, Gissot M, Croken MM, Kim K, et al (2011) *Toxoplasma gondii* sequesters centromeres to a specific nuclear region throughout the cell cycle. Proc Natl Acad Sci USA 108: 3767–3772. 21321216 10.1073/pnas.1006741108 PMC3048097

[39] Hu K (2008) Organizational changes of the daughter basal complex during the parasite replication of *Toxoplasma gondii* . PLOS Pathogens 4: e10. 18208326 10.1371/journal.ppat.0040010 PMC2211554

[40] Gubbels M-J, Vaishnava S, Boot N, Dubremetz J-F, Striepen B (2006) A MORN-repeat protein is a dynamic component of the *Toxoplasma gondii* cell division apparatus. J Cell Sci 119: 2236–2245. 16684814 10.1242/jcs.02949

[41] Lorestani A, Ivey FD, Thirugnanam S, Busby MA, Marth GT, et al (2012) Targeted proteomic dissection of *Toxoplasma* cytoskeleton sub-compartments using MORN1. Cytoskeleton (Hoboken) 69: 1069–1085. 23027733 10.1002/cm.21077 PMC3566231

[42] Sheiner L, Demerly JL, Poulsen N, Beatty WL, Lucas O, et al (2011) A systematic screen to discover and analyze apicoplast proteins identifies a conserved and essential protein import factor. PLOS Pathog 7: e1002392. 22144892 10.1371/journal.ppat.1002392 PMC3228799

[43] Meissner M, Brecht S, Bujard H, Soldati D (2001) Modulation of myosin A expression by a newly established tetracycline repressor-based inducible system in Toxoplasma gondii. Nucleic Acids Res 29: E115. 11713335 10.1093/nar/29.22.e115 PMC92585

[44] Huynh M-H, Rabenau KE, Harper JM, Beatty WL, Sibley LD, et al (2003) Rapid invasion of host cells by *Toxoplasma* requires secretion of the MIC2–M2AP adhesive protein complex. EMBO J 22: 2082–2090. 12727875 10.1093/emboj/cdg217 PMC156089

[45] Kafsack B, Beckers C, Carruthers VB (2004) Synchronous invasion of host cells by *Toxoplasma gondii* . Mol Biochem Parasitol 136: 309–311. 15478810 10.1016/j.molbiopara.2004.04.004

[46] Håkansson S, Charron AJ, Sibley LD (2001) *Toxoplasma* evacuoles: a two-step process of secretion and fusion forms the parasitophorous vacuole. EMBO J 20: 3132–3144. 11406590 10.1093/emboj/20.12.3132 PMC150190

[47] Mital J, Meissner M, Soldati D, Ward GE (2005) Conditional expression of *Toxoplasma gondii* apical membrane antigen-1 (TgAMA1) demonstrates that TgAMA1 plays a critical role in host cell invasion. Mol Biol Cell 16: 4341–4349. 16000372 10.1091/mbc.E05-04-0281 PMC1196342

[48] Carruthers VB, Giddings OK, Sibley LD (1999) Secretion of micronemal proteins is associated with *Toxoplasma* invasion of host cells. Cell Microbiol 1: 225–235. 11207555 10.1046/j.1462-5822.1999.00023.x

[49] Wiersma HI, Galuska SE, Tomley FM, Sibley LD, Liberator PA, et al (2004) A role for coccidian cGMP-dependent protein kinase in motility and invasion. Int J Parasitol 34: 369–380. 15003497 10.1016/j.ijpara.2003.11.019

[50] Lourido S, Shuman J, Zhang C, Shokat KM, Hui R, et al (2010) Calcium-dependent protein kinase 1 is an essential regulator of exocytosis in *Toxoplasma* . Nature 465: 359–362. 20485436 10.1038/nature09022 PMC2874977

[51] Billker O, Lourido S, Sibley LD (2009) Calcium-dependent signaling and kinases in apicomplexan parasites. Cell Host Microbe 5: 612–622. 19527888 10.1016/j.chom.2009.05.017 PMC2718762

[52] Nishi M, Hu K, Murray JM, Roos DS (2008) Organellar dynamics during the cell cycle of *Toxoplasma gondii* . J Cell Sci 121: 1559–1568. 18411248 10.1242/jcs.021089 PMC6810632

[53] Gubbels M-J, White M, Szatanek T (2008) The cell cycle and *Toxoplasma gondii* cell division: tightly knit or loosely stitched? Int J Parasitol 38: 1343–1358. 18703066 10.1016/j.ijpara.2008.06.004

[54] Striepen B, Crawford MJ, Shaw MK, Tilney LG, Seeber F, et al (2000) The plastid of *Toxoplasma gondii* is divided by association with the centrosomes. J Cell Biol 151: 1423–1434. 11134072 10.1083/jcb.151.7.1423 PMC2150670

[55] Vaishnava S, Morrison DP, Gaji RY, Murray JM, Entzeroth R, et al (2005) Plastid segregation and cell division in the apicomplexan parasite Sarcocystis neurona. J Cell Sci 118: 3397–3407. 16079283 10.1242/jcs.02458

[56] Chen C-T, Gubbels M-J (2013) The *Toxoplasma gondii* centrosome is the platform for internal daughter budding as revealed by a Nek1 kinase mutant. J Cell Sci 126: 3344–3355. 23729737 10.1242/jcs.123364 PMC3730244

[57] Francia ME, Jordan CN, Patel JD, Sheiner L, Demerly JL, et al (2012) Cell division in apicomplexan parasites is organized by a homolog of the striated rootlet fiber of algal flagella. PLOS Biology 10: e1001444. 23239939 10.1371/journal.pbio.1001444 PMC3519896

[58] Farrell M, Gubbels M-J (2014) The *Toxoplasma gondii* kinetochore is required for centrosome association with the centrocone (spindle pole). Cellular Microbiol 16: 78–94. 10.1111/cmi.12185 PMC3933516 24015880

[59] Francia ME, Striepen B (2014) Cell division in apicomplexan parasites. Nat Rev Microbiol 12: 125–136. 24384598 10.1038/nrmicro3184

[60] El-Haddad H, Przyborski JM, Kraft LGK, McFadden GI, Waller RF, et al (2013) Characterization of Ttalv2, an essential charged repeat motif protein of the Tetrahymena thermophila membrane skeleton. Eukaryotic Cell 12: 932–940. 23606287 10.1128/EC.00050-13 PMC3675985

[61] Ren J, Wen L, Gao X, Jin C, Xue Y, et al (2008) CSS-Palm 2.0: an updated software for palmitoylation sites prediction. Protein Eng Des Sel 21: 639–644. 18753194 10.1093/protein/gzn039 PMC2569006

[62] Fung C, Beck JR, Robertson SD, Gubbels M-J, Bradley PJ (2012) *Toxoplasma* ISP4 is a central IMC sub-compartment protein whose localization depends on palmitoylation but not myristoylation. Mol Biochem Parasitol 184: 99–108. 22659420 10.1016/j.molbiopara.2012.05.002 PMC3383393

[63] Beck JR, Rodriguez-Fernandez IA, de Leon JC, Huynh M-H, Carruthers VB, et al (2010) A novel family of *Toxoplasma* IMC proteins displays a hierarchical organization and functions in coordinating parasite division. PLOS Pathog 6: e1001094. 20844581 10.1371/journal.ppat.1001094 PMC2936552

[64] Lourido S, Tang K, Sibley LD (2012) Distinct signalling pathways control *Toxoplasma* egress and host-cell invasion. EMBO J 31: 4524–4534. 23149386 10.1038/emboj.2012.299 PMC3545288

[65] McCoy JM, Whitehead L, van Dooren GG, Tonkin CJ (2012) TgCDPK3 regulates calcium-dependent egress of *Toxoplasma gondii* from host cells. PLOS Pathog 8: e1003066. 23226109 10.1371/journal.ppat.1003066 PMC3514314

[66] Mann T, Beckers C (2001) Characterization of the subpellicular network, a filamentous membrane skeletal component in the parasite *Toxoplasma gondii* . Mol Biochem Parasitol 115: 257–268. 11420112 10.1016/s0166-6851(01)00289-4

[67] Brossier F, David Sibley L (2005) *Toxoplasma gondii*: microneme protein MIC2. Int J Biochem Cell Biol 37: 2266–2272. 16084754 10.1016/j.biocel.2005.06.006

[68] Huynh M-H, Carruthers VB (2006) *Toxoplasma* MIC2 is a major determinant of invasion and virulence. PLOS Pathog 2: e84. 16933991 10.1371/journal.ppat.0020084 PMC1550269

[69] Kessler H, Herm-Götz A, Hegge S, Rauch M, Soldati-Favre D, et al (2008) Microneme protein 8—a new essential invasion factor in *Toxoplasma gondii* . J Cell Sci 121: 947–956. 18319299 10.1242/jcs.022350

[70] Giovannini D, Spath S, Lacroix C, Perazzi A, Bargieri D, et al (2011) Independent roles of apical membrane antigen 1 and rhoptry neck proteins during host cell invasion by apicomplexa. Cell Host Microbe 10: 591–602. 22177563 10.1016/j.chom.2011.10.012

[71] Kremer K, Kamin D, Rittweger E, Wilkes J, Flammer H, et al (2013) An overexpression screen of *Toxoplasma gondii* RabGTPases reveals distinct transport routes to the micronemes. PLOS Pathog 9: e1003213. 23505371 10.1371/journal.ppat.1003213 PMC3591302

[72] Andenmatten N, Egarter S, Jackson AJ, Jullien N, Herman J-P, et al (2012) Conditional genome engineering in *Toxoplasma gondii* uncovers alternative invasion mechanisms. Nat Methods 10: 125–127. 23263690 10.1038/nmeth.2301 PMC3605914

[73] Nagamune K, Moreno SN, Chini EN, Sibley LD (2008) Calcium regulation and signaling in apicomplexan parasites. Subcell Biochem 47: 70–81. 18512342 10.1007/978-0-387-78267-6_5

[74] Hopp CS, Bowyer PW, Baker DA (2012) The role of cGMP signalling in regulating life cycle progression of Plasmodium. Microbes Infect 14: 831–837. 22613210 10.1016/j.micinf.2012.04.011 PMC3484397

[75] Blackman MJ, Carruthers VB (2013) Recent insights into apicomplexan parasite egress provide new views to a kill. Curr Opin Microbiol 16: 459–464. 23725669 10.1016/j.mib.2013.04.008 PMC3755044

[76] Paredes-Santos TC, de Souza W, Attias M (2012) Dynamics and 3D organization of secretory organelles of *Toxoplasma gondii* . J Struct Biol 177: 420–430. 22155668 10.1016/j.jsb.2011.11.028

[77] Treeck M, Sanders JL, Elias JE, Boothroyd JC (2011) The phosphoproteomes of *Plasmodium falciparum* and *Toxoplasma gondii* reveal unusual adaptations within and beyond the parasites' boundaries. Cell Host Microbe 10: 410–419. 22018241 10.1016/j.chom.2011.09.004 PMC3254672

[78] Nebl T, Prieto JH, Kapp E, Smith BJ, Williams MJ, et al (2011) Quantitative in vivo analyses reveal calcium-dependent phosphorylation sites and identifies a novel component of the *Toxoplasma* invasion motor complex. PLOS Pathogens 7: e1002222. 21980283 10.1371/journal.ppat.1002222 PMC3182922

[79] Okamoto N, Keeling PJ (2014) The 3D structure of the apical complex and association with the flagellar apparatus revealed by serial TEM tomography in *Psammosa* pacifica, a distant relative of the Apicomplexa. PLOS ONE 9: e84653. 24392150 10.1371/journal.pone.0084653 PMC3879320

[80] Obornik M, Vancová M, Lai D-H, Janouškovec J, Keeling PJ, et al (2011) Morphology and ultrastructure of multiple life cycle stages of the photosynthetic relative of apicomplexa, Chromera velia. Protist 162: 115–130. 20643580 10.1016/j.protis.2010.02.004

[81] Okamoto N, Horák A, Keeling PJ (2012) Description of two species of early branching dinoflagellates, *Psammosa* pacifica n. g., n. sp. and P. atlantica n. sp. PLOS ONE 7: e34900. 22719825 10.1371/journal.pone.0034900 PMC3377698

[82] Perkins FO (1996) The structure of Perkinsus marinus (Mackin, Owen and Collier, 1950) Levine, 1978 with comments on taxonomy and phylogeny of Perkinsus spp. J Shellfish Res 15: 67–87.

[83] Portman N, Foster C, Walker G, Šlapeta J (2014) Evidence of intraflagellar transport and apical complex formation in a free-living relative of the apicomplexa. Eukaryotic Cell 13: 10–20. 24058169 10.1128/EC.00155-13 PMC3910950

[84] Johnson J-LF, Leroux MR (2010) cAMP and cGMP signaling: sensory systems with prokaryotic roots adopted by eukaryotic cilia. Trends Cell Biol 20: 435–444. 20541938 10.1016/j.tcb.2010.05.005

[85] Striepen B, Soldati D (2007) Genetic manipulation of *Toxoplasma gondii* In: Weiss LM, Kim K, editors. *Toxoplasma gondii*. The Model Apicomplexan-Perspectives and Methods. London: Elsevier. pp. 391–415.

[86] van Dooren GG, Tomova C, Agrawal S, Humbel BM, Striepen B (2008) *Toxoplasma gondii* Tic20 is essential for apicoplast protein import. Proc Natl Acad Sci USA 105: 13574–13579. 18757752 10.1073/pnas.0803862105 PMC2533231

[87] van Dooren GG, Reiff SB, Tomova C, Meissner M, Humbel BM, et al (2009) A novel dynamin-related protein has been recruited for apicoplast fission in *Toxoplasma gondii* . Curr Biol 19: 267–276. 19217294 10.1016/j.cub.2008.12.048 PMC3941992

[88] McMillan PJ, Millet C, Batinovic S, Maiorca M, Hanssen E, et al (2013) Spatial and temporal mapping of the PfEMP1 export pathway in Plasmodium falciparum. Cell Microbiol 15: 1401–1418. 23421990 10.1111/cmi.12125 PMC3711974

