# Supplementary material for: The Apical Complex Provides a Regulated Gateway for Secretion of Invasion Factors in Toxoplasma
Source: PLoS Pathog. 2014 Apr 17;10(4):e1004074. doi: 10.1371/journal.ppat.1004074 (PMC3990729; doi:10.1371/journal.ppat.1004074)
Supplement: Figure S1 — Live cell imaging of the N- and C-termini of RNG2. (A-B) A Western blot depicting mCherry-cMyc-RNG2-GFP parasites probed with (A) anti-cMyc and (B) anti-GFP. In both blots, the masses of the tagged RNG2 protein are equivalent in size (>260 kDa), indicative of successful targeting of both termini of the gene. (C) Live cell imaging of mCherry-cMyc-RNG2-GFP intracellular parasites. Arrowheads depict the apical ring. In all parasites, N-terminal mCherry labeling is posterior to the C-terminal GFP labeling. (i) In the newly formed apical rings of daughter buds, the N-terminus of RNG2 is also posterior to the C-terminus (bottom panel, arrows). Note: fluorescence is also retained in the residual bodies. (D) Live cell imaging of mCherry-c-myc-RNG2-GFP parasites in extracellular parasites. (i) N-terminal mCherry labeling is posterior to the C-terminal GFP-labeling when the conoid is retracted. (ii) Treatment with Ca2+ ionophore A23187 causes conoid extrusion, and relocation of the N-terminus of RNG2 to the anterior side of the C-terminus. Scale bars are 2 μm. (PDF) [file ppat.1004074.s001.pdf]

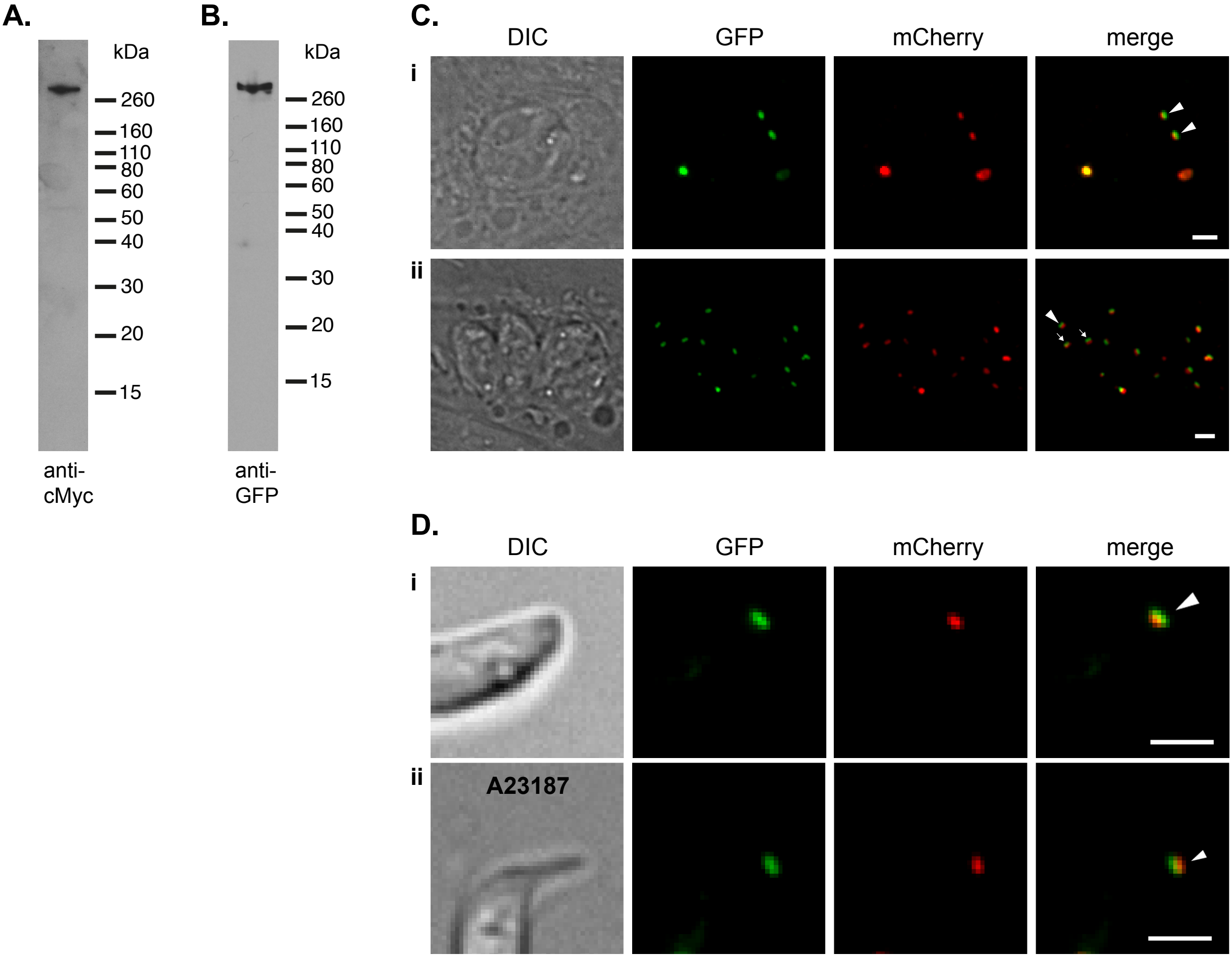

Figure S1. Live cell imaging of the N- and C-termini of RNG2. (A, B) A western blot depicting mCherry-c-myc-RNG2-GFP parasites probed with (A) anti-c-myc and (B) anti-GFP. In both blots, the masses of the tagged RNG2 protein are equivalent in size (>260 kDa), indicative of successful targeting of both termini of the gene. (C) Live cell imaging of mCherry-c-myc-RNG2-GFP parasites in intracellular parasites. Arrowheads depict the apical ring. In all parasites, N-terminal mCherry labelling is posterior to the C-terminal GFP labelling. In the newly formed apical rings of daughter buds, the N-terminus of RNG2 is also posterior to the C-terminus (bottom panel, arrows). (D) Live cell imaging of mCherry-c-myc-RNG2-GFP in extracellular parasites. N-terminal mCherry labelling is posterior to the C-terminal GFP-labelling. In addition to the apical ring, we observed labelling of an additional structure in extracellular parasites. The identity of this structure and the relevance of this RNG2 localisation remain unclear. It is possible that this structure represents an artefact of adding bulky tags to RNG2. (E) Treatment with Ca2+ ionophore A23187 causes conoid extrusion, and relocation of the N-terminus of RNG2 to the anterior side of the C-terminus. Scale bars are 2 μm.
