# Supplementary material for: The Apical Complex Provides a Regulated Gateway for Secretion of Invasion Factors in Toxoplasma
Source: PLoS Pathog. 2014 Apr 17;10(4):e1004074. doi: 10.1371/journal.ppat.1004074 (PMC3990729; doi:10.1371/journal.ppat.1004074)
Supplement: Figure S2 — Western blot and immunofluorescence assays for microneme maturity with RNG2 depletion. (A) In parasite total protein samples, microneme proteins MIC2 and AMA1, and mitochondrial protein Tom40, show equivalent amounts of protein in iΔHA-RNG2 cells treated with or without ATc for three days. Only HA-RNG2, detected by HA antibodies, shows depletion with ATc treatment. Equal cell numbers were used in all gel lanes. (B, C) IFA detection of (B) AMA1 and HA-RNG2, or (C) MIC2 in intracellular iΔHA-RNG2 cells treated with or without ATc for two days. Scale bar = 5 μm. (PDF) [file ppat.1004074.s002.pdf]

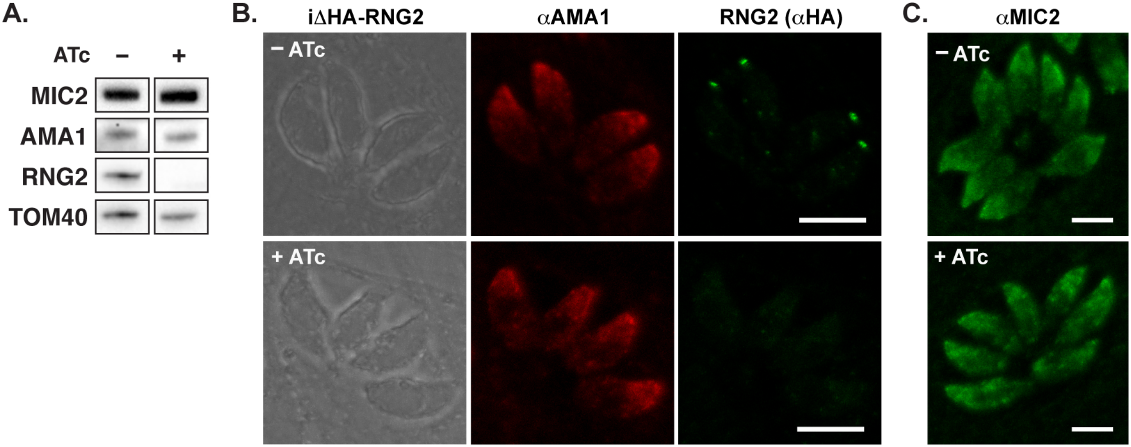

Figure S2. Western blot and immunofluorescence assays for microneme maturity with RNG2 depletion. (A) In parasite total protein samples, microneme proteins MIC2 and AMA1, and mitochondrial protein Tom40, show equivalent amounts of protein in iΔHA-RNG2 cells treated with or without ATc for three days. Only HA-RNG2, detected by HA antibodies, shows depletion with ATc treatment. Equal cell numbers were used in all gel lanes. (B, C) IFA detection of (B) AMA1 and HA-RNG2, or (C) MIC2 in intracellular iΔHA-RNG2 cells treated with or without ATc for two days. Scale bar = 5 μm.
