# Supplementary material for: The Apical Complex Provides a Regulated Gateway for Secretion of Invasion Factors in Toxoplasma
Source: PLoS Pathog. 2014 Apr 17;10(4):e1004074. doi: 10.1371/journal.ppat.1004074 (PMC3990729; doi:10.1371/journal.ppat.1004074)
Supplement: Figure S3 — 8-Br-cGMP-stimulation of microneme secretion is muted in RNG2 minus cells. MIC2 secretion without RNG2 (iΔHA-RNG2 cells +ATc) or with RNG2 (iΔHA-RNG2 cells -ATc and parental cells). Constitutive MIC2 secretion, and secretion with exogenous cGMP (by analogue 8-Br-cGMP) (A), or calcium stimulation (by ionophore A23187) (B) is assayed by Western blot. Stimulated microneme secretion by exogenous calcium is strong in all cells, but by exogenous cGMP is reduced in RNG2 knockdown cells. (PDF) [file ppat.1004074.s003.pdf]

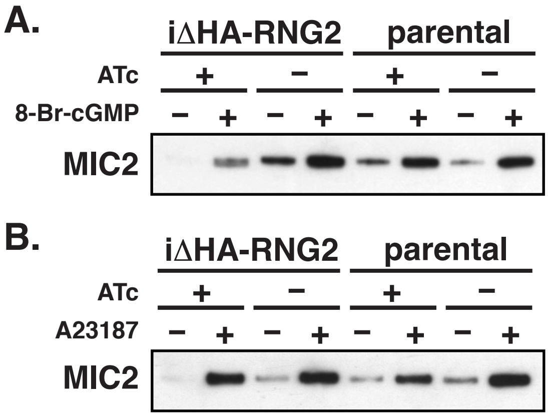

Figure S3. 8-Br-cGMP-stimulation of microneme secretion is muted in RNG2 minus cells. MIC2 secretion without RNG2 (iΔHA-RNG2 cells +ATc) or with RNG2 (iΔHA-RNG2 cells -ATc and parental cells). Constitutive MIC2 secretion, and secretion with exogenous cGMP (by analogue 8-Br-cGMP) (A), or calcium stimulation (by ionophore A23187) (B) is assayed by western blot. Stimulated microneme secretion by exogenous calcium is strong in all cells, but by exogenous cGMP is reduced in RNG2 knockdown cells.
