# Supplementary material for: The Apical Complex Provides a Regulated Gateway for Secretion of Invasion Factors in Toxoplasma
Source: PLoS Pathog. 2014 Apr 17;10(4):e1004074. doi: 10.1371/journal.ppat.1004074 (PMC3990729; doi:10.1371/journal.ppat.1004074)
Supplement: Table S1 — Antibodies used for microscopy and protein assays. (PDF) [file ppat.1004074.s004.pdf]

Antibody	Dilution	Application	Source
Rat anti-HA	1/100	IFA	Roche
	1/1000	Western blot	
Mouse anti c-myc	1/200	IFA	Roche
	1/500	Western blot	
	1/200	Western blot	Santa Cruz
Mouse anti-IMC1	1/500	IFA	Gary Ward (University of Vermont, USA)
Rabbit anti-GAP45	1/500	IFA	Dominique Soldati-Favre (University of Geneva, Switzerland)
Rabbit anti-Centrin1	1/500	IFA	Abcam
Mouse anti-tubulin	1/100	IFA	mAb 12G10, Developmental Studies Hybridoma Bank, University of Iowa
Mouse anti-MIC2 clone 6D10	1/1000	Western blot	David Sibley (Washington University, USA)
Mouse anti-AMA1 clone B3.93	1/1000	Western blot	Chris Tonkin (WEHI, Australia)
Rabbit anti-Tom40	1/1000	Western blot	Giel van Dooren (Australian National University, Australia)
Mouse anti-ROP1	1/1000	IFA	John Boothroyd (Stanford University, USA)
Mouse anti-SAG1	1/1000	IFA	Abcam
Mouse anti-GFP	1/500	Western blot	Roche
Mouse anti-GRA8	1/500	Western blot	Gary Ward (University of Vermont, USA)
Mouse anti-AMA1 CL.22	1/500	Western blot	Chris Tonkin (WEHI, Australia)

Table S1: Antibodies used for microscopy and protein assays
